# Supplementary material for: Therapies for Long COVID in non-hospitalised individuals: from symptoms, patient-reported outcomes and immunology to targeted therapies (The TLC Study)
Source: BMJ Open. 2022 Apr 25;12(4):e060413. doi: 10.1136/bmjopen-2021-060413 (PMC9044550; doi:10.1136/bmjopen-2021-060413)
Supplement: Author's manuscript [file bmjopen-2021-060413.draft_revisions.pdf]

BMJ Open is committed to open peer review. As part of this commitment we make the peer review history of every article we publish publicly available.

When an article is published we post the peer reviewers' comments and the authors' responses online. We also post the versions of the paper that were used during peer review. These are the versions that the peer review comments apply to.

The versions of the paper that follow are the versions that were submitted during the peer review process. They are not the versions of record or the final published versions. They should not be cited or distributed as the published version of this manuscript.

BMJ Open is an open access journal and the full, final, typeset and author-corrected version of record of the manuscript is available on our site with no access controls, subscription charges or pay-per-view fees (http://bmjopen.bmj.com).

If you have any questions on BMJ Open's open peer review process please email info.bmjopen@bmj.com

BMJ Open

# Therapies for Long COVID in non-hospitalised individuals - from symptoms, patient-reported outcomes, and immunology to targeted therapies (The TLC Study): Study protocol



| | |
|---|---|
| Journal: | *BMJ Open* |
| Manuscript ID | bmjopen-2021-060413 |
| Article Type: | Protocol |
| Date Submitted by the Author: | 21-Dec-2021 |





| | |
|---|---|
| Complete List of Authors: | Haroon, Shamil; University of Birmingham, Institute of Applied Health Research<br>Nirantharakumar, Krishnarajah; University of Birmingham, Institute of Applied Health Research; Midlands Health Data Research UK<br>Hughes, Sarah; University of Birmingham, Institute of Applied Health Research; National Institute for Health Research (NIHR) Applied Research Centre West Midlands<br>Subramanian, Anuradhaa; University of Birmingham, Institute of Applied Health Research<br>Aiyegbusi, Olalekan Lee; University of Birmingham, Institute of Applied Health Research; National Institute for Health Research (NIHR) Applied Research Centre West Midlands<br>Davies, Elin Haf; Aparito Ltd<br>Myles, Puja; Medicines and Healthcare Products Regulatory Agency<br>Williams, Tim; Medicines and Healthcare Products Regulatory Agency<br>Turner, Grace; University of Birmingham, Institute of Applied Health Research<br>Chandan, Joht Singh; University of Birmingham, Institute of Applied Health Research<br>McMullan, Christel; University of Birmingham, Institute of Applied Health Research<br>Lord, Janet; University of Birmingham, Institute of Inflammation and Ageing<br>Wraith, David; University of Birmingham, Institute of Immunology and Immunotherapy<br>McGee, Kirsty; University of Birmingham, Institute of Inflammation and Ageing<br>Denniston, Alastair; University Hospitals Birmingham NHS Foundation Trust<br>Taverner, Thomas ; University of Birmingham, Institute of Applied Health Research<br>Jackson, Louise; University of Birmingham, Institute of Applied Health Research<br>Sapey, Elizabeth; University of Birmingham, Institute of Inflammation and Ageing<br>Gkoutos, George; University of Birmingham, Institute of Cancer and Genomic Sciences<br>Gokhale, Krishna; University of Birmingham, Institute of Applied Health |

| | |
|---|---|
| | Research<br>Leggett, Edward; Medicines and Healthcare Products Regulatory Agency, CPRD<br>Iles, Clare; Medicines and Healthcare Products Regulatory Agency<br>Frost, Christopher; Aparito Ltd<br>McNamara, Gary; Aparito Ltd<br>Bamford, Amy; University Hospitals Birmingham NHS Foundation Trust<br>Marshall, Tom; University of Birmingham, Institute of Applied Health Research<br>Zemedikun, Dawit; University of Birmingham, Institute of Applied Health Research<br>Price, Gary; Not applicable<br>Marwaha, Steven; University of Birmingham, Institute for Mental Health<br>Simms-Williams, Nikita; University of Birmingham, Institute of Applied Health Research<br>Brown, Kirsty; University of Birmingham, Institute of Applied Health Research<br>Walker, Anita; University of Birmingham, Institute of Applied Health Research<br>Jones, Karen; University of Birmingham, Institute of Applied Health Research<br>Matthews, Karen; Not applicable<br>Camaradou, Jennifer; Not applicable<br>Saint-Cricq, Michael; Not applicable<br>Kumar, Sumita; Not applicable<br>Alder, Yvonne; Not applicable<br>Stanton, David E.; Not applicable<br>Agyen, Lisa; Not applicable<br>Baber, Megan; Not applicable<br>Blaize, Hannah; Not applicable<br>Calvert , Melanie ; University of Birmingham, Institute of Applied Health Research; Midlands Health Data Research UK |


| Keywords: | COVID-19, IMMUNOLOGY, PUBLIC HEALTH, THERAPEUTICS |
|---|---|
| | |


SCHOLARONE™
Manuscripts

# Therapies for Long COVID in non-hospitalised individuals - from symptoms, patient-reported outcomes, and immunology to targeted therapies (The TLC Study): Study protocol

Authors: Shamil Haroon[1] (s.haroon@bham.ac.uk), Krishnarajah Nirantharakumar[1,2] (K.Nirantharan@bham.ac.uk), Sarah E Hughes[1,3,4] (S.E.Hughes@bham.ac.uk), Anuradhaa Subramanian[1] (A.Subramanian@bham.ac.uk), Olalekan Lee Aiyegbusi[1,3,4,5,6] (O.L.Aiyegbusi@bham.ac.uk), Elin Haf Davies[7] (elin@aparito.com), Puja Myles[8] (Puja.Myles@mhra.gov.uk), Tim Williams[8] (Tim.Williams@mhra.gov.uk), Grace M Turner[1,3] (G.Turner.1@bham.ac.uk), Joht Singh Chandan[1] (J.S.Chandan.1@bham.ac.uk), Christel McMullan[1,3] (C.McMullan@bham.ac.uk), Janet Lord[9] (J.M.LORD@bham.ac.uk), David Wraith[10] (D.Wraith@bham.ac.uk), Kirsty McGee[9] (K.C.McGee@bham.ac.uk), Alastair Denniston[11] (A.Denniston@bham.ac.uk), Tom Taverner[1] (T.Taverner@bham.ac.uk), Louise Jackson[1] (L.Jackson.1@bham.ac.uk), Elizabeth Sapey[9] (E.Sapey@bham.ac.uk), Georgios Gkoutos[12] (G.Gkoutos@bham.ac.uk), Krishna Gokhale[1] (K.M.Gokhale@bham.ac.uk), Edward Leggett[8] (Edward.Leggett@mhra.gov.uk), Clare Iles[8] (Clare.Iles@mhra.gov.uk), Christopher Frost[7] (chris.frost@aparito.com), Gary McNamara[7] (gary.mcnamara@aparito.com), Amy Bamford[11] (Amy.Bamford@uhb.nhs.uk), Tom Marshall[1] (T.P.MARSHALL@bham.ac.uk), Dawit Zemedikun[1] (D.T.Zemedikun@bham.ac.uk), Gary Price (tlcstudy@bham.ac.uk), Steven Marwaha[13] (S.Marwaha@bham.ac.uk), Nikita Simms-Williams[1] (N.Simms-Williams@bham.ac.uk), Kirsty Brown[1] (BrownKR@adf.bham.ac.uk), Anita Walker[1] (A.A.Walker@bham.ac.uk), Karen Jones[1] (K.Jones.2@bham.ac.uk), Karen Matthews (tlcstudy@bham.ac.uk), Jennifer Camaradou (tlcstudy@bham.ac.uk), Michael Saint-Cricq (tlcstudy@bham.ac.uk), Sumita Kumar (tlcstudy@bham.ac.uk), Yvonne Alder (tlcstudy@bham.ac.uk), David E. Stanton (tlcstudy@bham.ac.uk), Lisa Agyen (tlcstudy@bham.ac.uk), Megan Baber (tlcstudy@bham.ac.uk), Hannah Blaize (tlcstudy@bham.ac.uk), Melanie Calvert[1,2,3,4,5,6] (M.Calvert@bham.ac.uk)

Corresponding author: Shamil Haroon

Institute of Applied Health Research,
College of Medical and Dental Sciences,

For peer review only - http://bmjopen.bmj.com/site/about/guidelines.xhtml

University of Birmingham,
Edgbaston,
Birmingham,
B15 2TT
T: +44 (0) 121 414 5042

## Affiliations

1. Institute of Applied Health Research, University of Birmingham, Birmingham, UK
2. Midlands Health Data Research UK, Birmingham, UK
3. Centre for Patient Reported Outcomes Research, Institute of Applied Health Research, University of Birmingham, Birmingham, UK
4. National Institute for Health Research (NIHR) Applied Research Centre West Midlands, Birmingham, UK
5. Birmingham Health Partners Centre for Regulatory Science and Innovation, University of Birmingham, Birmingham, UK
6. NIHR Birmingham Biomedical Research Centre, NIHR Surgical Reconstruction and Microbiology Research Centre, University of Birmingham, Birmingham, UK
7. Aparito Ltd, Wrexham, UK
8. Clinical Practice Research Datalink, Clinical Practice Research Datalink, Medicines and Healthcare Products Regulatory Agency, London, UK
9. Institute of Inflammation and Ageing, University of Birmingham, Birmingham, UK
10. Institute of Immunology and Immunotherapy, University of Birmingham, Birmingham UK
11. University Hospitals Birmingham NHS Foundation Trust, Birmingham, UK
12. Institute of Cancer and Genomic Sciences, University of Birmingham, Birmingham, UK
13. Institute for Mental Health, University of Birmingham, Birmingham, UK

## Author contributions

SH and MC oversaw and contributed to the drafting of the entire manuscript with input from all co-authors. KN, AS, AD, TT, GG provided methodological and statistical expertise. JSC and OLA supported drafting the background and co-led the evidence review on post-viral syndromes. OLA was academic lead for patient and public involvement. KM, GP, JC, MSC, SK, YA, DES, LA, MB and HB provided expert patient input based on their lived experience of COVID and its long-term effects. SEH, MC and CM led the development of the Symptom Burden Questionnaire ™ for Long COVID. PM, TW, CI and EL provided expertise on CPRD Aurum data and the Interventional Research Service Platform. EHD and CF provided input on the use of Atom5™, ePROs and wearables. JL and DW provided subject and methodological expertise on the immunological analyses with input from KM. DZ and LJ drafted the sections on health econometrics. GT drafted the section on the over-the-counter medication survey. ES, AB, SM and TM provided clinical and public health expertise. GK supported data extraction from CPRD Aurum and is the creator of DExTER. KB and NSW supported revisions to the manuscript and referencing. AW and KJ provided administrative support for the project. AW assisted with usability testing and protocol development. All co-authors reviewed and approved the manuscript.

For peer review only

## Funding statement

This work is independent research jointly funded by the National Institute for Health Research (NIHR) and UK Research and Innovation (UKRI) (Therapies for Long COVID in non-hospitalised individuals: From symptoms, patient reported outcomes and immunology to targeted therapies (The TLC Study), COV-LT-0013). The views expressed in this publication are those of the author(s) and not necessarily those of the National Institute for Health Research or the Department of Health and Social Care.


## Conflicts of interest

MC is Director of the Birmingham Health Partners Centre for Regulatory Science and Innovation, Director of the Centre for the Centre for Patient Reported Outcomes Research and is a National Institute for Health Research (NIHR) Senior Investigator. MC receives funding from the National Institute for Health Research (NIHR) Birmingham Biomedical Research Centre, the NIHR Surgical Reconstruction and Microbiology Research Centre and NIHR ARC West Midlands at the University of Birmingham and University Hospitals Birmingham NHS Foundation Trust, Health Data Research UK, Innovate UK (part of UK Research and Innovation), Macmillan Cancer Support, UCB Pharma, Janssen, GSK and Gilead. MC has received personal fees from Astellas, Aparito Ltd, CIS Oncology, Takeda, Merck, Daiichi Sankyo, Glaukos, GSK and the Patient-Centered Outcomes Research Institute (PCORI) outside the submitted work.

SEH is supported by the National Institute for Health Research (NIHR) Applied Research Centre (ARC), West Midlands at the University of Birmingham. SEH has received personal fees from Cochlear Ltd and Aparito Ltd outside the submitted work.

OLA receives funding from the NIHR Birmingham Biomedical Research Centre (BRC), NIHR Applied Research Centre (ARC), West Midlands at the University of Birmingham and University Hospitals Birmingham NHS Foundation, Innovate UK (part of UK Research and Innovation), Gilead Sciences Ltd, and Janssen pharmaceuticals, Inc. OLA declares personal fees from Gilead Sciences Ltd, GlaxoSmithKline (GSK) and Merck outside the submitted work.

CM receives funding from the National Institute for Health Research (NIHR) Surgical Reconstruction and Microbiology Research Centre, Innovate UK, and has received personal fees from Aparito Ltd outside the submitted work.

PM, TW, CI and EL are employees of CPRD, the data custodians for CPRD Aurum. CPRD is jointly sponsored by the UK government's Medicines and Healthcare products Regulatory Agency and the National Institute for Health Research (NIHR). As a not-for-profit UK government body, CPRD seeks to recoup the cost of delivering its research services to academic, industry and government researchers through research user license fees.

JC is a lay member on the UK NICE COVID expert panel, a citizen partner to the COVID END Evidence Synthesis Network, PPI lead on the NIHR CICADA ME Study, patient representative at the EAN European Neurology Autonomic Nervous Systems Disorders Working Group, a member of the MRC/UKRI Advanced Pain Discovery Platform, and external board member of Plymouth Institute of Health. She also reports contracts with GSK and Medable.

All other co-authors have no relevant competing interests to declare.

For peer review only

## Data statement

Data for this project are not currently available for access outside the research team. Each of the datasets generated within the project may be shared when finalised but this will require an application to the data controllers. The data may then be released to a specific research team for a specific project dependent on the independent approvals being in place.

## Key words

Long COVID, post-COVID-19 syndrome, symptoms, electronic health records, therapies

## Word count

3961

For peer review only

# Abstract

## Introduction

Individuals with COVID-19 frequently experience symptoms and impaired quality of life beyond 4-12 weeks, commonly referred to as Long COVID. Whether Long COVID is one or several distinct syndromes is unknown. Establishing the evidence base for appropriate therapies is needed. We aim to evaluate the symptom burden and underlying pathophysiology of Long COVID syndromes in non-hospitalised individuals and evaluate potential therapies.

## Methods and analysis

A cohort of 4000 non-hospitalised individuals with a past COVID-19 diagnosis and 1000 matched controls will be selected from anonymised primary care records from the Clinical Practice Research Datalink (CPRD) and invited by their general practitioners to participate on a digital platform (Atom5™). Individuals will report symptoms, quality of life, work capability, and patient reported outcome measures. Data will be collected monthly for one year.

Statistical clustering methods will be used to identify distinct Long COVID symptom clusters. Individuals from the four most prevalent clusters and two control groups will be invited to participate in the BioWear sub-study which will further phenotype Long COVID symptom clusters by measurement of immunological parameters and actigraphy.

We will review existing evidence on interventions for post-viral syndromes and Long COVID to map and prioritise interventions for each newly characterised Long COVID syndrome. Recommendations will be made using the cumulated evidence in an expert consensus workshop. A virtual supportive intervention will be coproduced with patients and health service providers for future evaluation.

Individuals with lived experience of Long COVID will be involved throughout this programme through a patient and public involvement group.

## Ethics and dissemination

Ethical approval was obtained from the Solihull Research Ethics Committee, West Midlands (21/WM/0203). The study is registered on the ISRCTN Registry (1567490). Research findings will be presented at international conferences, in peer-reviewed journals, to Long COVID patient support groups and to policymakers.


For peer review only

# Article Summary

## Strengths and limitations of the study

- The study will generate a nationally representative cohort of individuals with Long COVID recruited from primary care.
- We will recruit controls matched on a wide range of demographic and clinical factors to assess differences in symptoms between people with Long COVID and similar individuals without a history of COVID-19.
- We will use a newly developed electronic patient reported outcome measure (Symptom Burden Questionnaire™) for Long COVID to comprehensively assess a wide range of symptoms highlighted by existing literature, patients, and clinicians.
- Immunological, proteomic, genetic, and wearable data captured in the study will allow deep phenotyping of Long COVID syndromes to help better target therapies.
- A limitation is that a significant proportion of non-hospitalised individuals affected by COVID-19 in the first wave of the pandemic will lack confirmatory testing and will be excluded from recruitment to the study.

For peer review only

# Introduction

Coronavirus Disease 2019 (COVID-19), caused by Severe Acute Respiratory Syndrome Coronavirus 2 (SARS CoV-2), is the most significant pandemic since the Spanish Influenza Pandemic of 1918. There have been over 240 million cases worldwide, including over 8.4 million cases in the UK.[1,2] In the UK, this has resulted in over 550,000 hospital admissions and 160,000 deaths.[3,4]

SARS CoV-2 enters human cells using the angiotensin II receptor, which is widely expressed throughout the body. COVID-19 is consequently a multisystem disorder affecting the lungs, heart, gut, kidneys, brain, and skin. A wide range of symptoms have been associated with COVID-19 including breathlessness, fatigue, fever, myalgia, “brain fog” and anosmia.[5]

Symptoms resolve within 12 weeks in most affected individuals. However, in up to 10% of individuals, symptoms of COVID-19 persist, sometimes in a relapsing remitting manner.[6] This can have significant impacts on physical and neurocognitive functioning, quality of life and work capability.[5] Those having symptoms lasting beyond 12 weeks are referred to as experiencing post-COVID-19 syndrome (NICE 2019) or more widely known as Long COVID.[7]

The cause of Long COVID is poorly understood. However, there is evidence from other post-viral syndromes that may be applicable to Long COVID. Evidence from existing studies on COVID-19 suggest a significant burden of pathophysiological insults and sequelae such as lung scarring, kidney injury, myocarditis and systemic inflammatory states that may promulgate longer term symptoms.[5] It is possible that autoimmune pathways may be triggered by COVID-19, leading to multi-system inflammatory damage.[8]

Current evidence suggests that 2.3-37% of individuals with COVID-19 experience symptoms and impaired quality of life and frequently report experiencing heterogeneous physical and psychological symptoms beyond 12 weeks (“Long COVID”) that can be debilitating.[9–11] People living with Long COVID have indicated that they are suffering with a range of symptoms, feel ‘abandoned’ and ‘dismissed’ by healthcare providers and receive limited or conflicting advice.[12] The aetiology, pathophysiology and treatments for Long COVID are not well understood, creating an unmet need for the growing number of affected individuals. Although efforts are being made to study Long COVID in hospitalised patients, there is a large unmet need among non-hospitalised individuals. Long COVID may comprise several distinct syndromes yet to be fully characterised.

Existing evidence on Long COVID is based on hospitalised cohorts and non-selected populations that are unlikely to be generalisable to the wider UK population of non-hospitalised individuals. A representative population-based cohort with well-matched controls, ideally derived from primary care, is needed to understand the burden of Long COVID, associated disability and impact on work capability. Current literature suggests the physical and psychological symptoms of Long COVID are highly diverse and may represent several distinct syndromes.[13] The characterisation of these syndromes using real-world data in combination with patient reported outcomes would aid health services to deliver appropriate interventions to meet these health needs.

The aetiology and risk factors for Long COVID also need further investigation as literature suggests a disproportionately higher prevalence of Long COVID among women, older adults and individuals with specific symptom clusters.[11,14,15] This may be due to differing immunological profiles of individuals

For peer review only

who go on to develop Long COVID.[16,17] For example, older adults with COVID-19 show higher levels of senescent T cells.[18] As these are pro-inflammatory, more likely to be autoimmune and tissue-damaging, their persistence could mediate distinct symptoms in Long COVID among these individuals. Understanding the immunological basis of Long COVID would better enable clinicians to offer targeted therapies.[19]

There is an urgent need for evidence-based, accessible interventions, co-produced with patients with lived experience of COVID-19, to better support the growing number of non-hospitalised individuals with Long COVID. We propose to derive treatment recommendations drawing upon existing approaches to the management of post-viral syndromes in addition to a detailed understanding of Long COVID syndromes.

There is also a need to develop a trial platform that can link symptom, primary care, and hospital data with immunological profiling to evaluate interventions for Long COVID in non-hospitalised patients. The Recovery trial and PHOSP-COVID study have demonstrated the strength of platforms where several interventions can be rapidly evaluated in secondary care.[20,21] The PRINCIPLE study was set up for non-hospitalised patients in primary care with acute COVID-19.[22] However, such an infrastructure is still needed for affected individuals with Long COVID in the wider community, and studies such as STIMULATE-ICP are being set up to help address this.[23]

The TLC Study will directly address the patient needs highlighted in the NIHR Evidence Report, by the LongCovidSOS campaign, our patient and public involvement group and patient partners, who have co-designed this study.[24,25] Our research will characterise the symptoms, health impacts and underlying pathophysiology of Long COVID, define its component syndromes in non-hospitalised individuals, and provide resources to support symptom management and nurse-led support for those with the severest symptoms. We will use our findings to co-produce with patients a targeted intervention for Long COVID, tailored to individual patient needs.

For peer review only

## Aims

The aim of the study is to evaluate the symptom burden and underlying pathophysiology of Long COVID syndromes in non-hospitalised individuals and the impact on quality of life and work capability. We also aim to identify potential therapies and co-produce a remotely delivered intervention for supporting individuals with Long COVID syndromes. These findings will be used to inform a feasibility study to evaluate the intervention produced during this project, and to plan future evaluations of supportive and pharmacological interventions for treating Long COVID. The methods for therapy identification, intervention development and feasibility testing will be reported in separate linked protocols.

The key objectives are:

1. To establish a representative population-based cohort of non-hospitalised individuals with COVID-19 and prolonged symptoms ("Long COVID").
2. To validate a new patient-reported outcome (PRO) measure of symptom burden in individuals with Long COVID- the Symptom Burden Questionnaire ™ for Long COVID.
3. To collect validated PROs including symptoms, quality of life and work capability data longitudinally from the representative cohort of non-hospitalised individuals with Long COVID.
4. To identify distinct symptom clusters ("syndromes") in individuals with Long COVID and compare these Long COVID syndromes to known post-viral syndromes.
5. To measure immunological, proteomic and miRNA biomarkers to determine the underlying pathogenesis associated with Long COVID syndromes. To capture physical health measures via a wearable device to determine the physical exertions (heart rate intensity and movement) and sleep patterns observed in Long COVID syndromes.
6. To make recommendations on pharmacological and supportive therapies that: i) should be implemented without need for further evaluation; ii) should be evaluated in a clinical trial, and iii) should not be recommended for treating long COVID based on the available evidence.
7. Co-produce a supportive, remotely delivered intervention for individuals with Long COVID, to address the symptoms associated with each Long COVID syndrome.
8. To evaluate patient and public involvement in the study.

For peer review only

# Methods and analysis

## Study design

We will undertake a population-based cohort study of non-hospitalised individuals with Long COVID (cases) and matched controls recruited from general practices in England that contribute data to the Clinical Practice Research Datalink (CPRD) Aurum database (Figure 1).[26] CPRD is a real-world research service supporting retrospective and prospective public health and clinical studies. CPRD is jointly sponsored by the Medicines and Healthcare products Regulatory Agency and the National Institute for Health Research (NIHR), as part of the Department of Health and Social Care. CPRD collects anonymised patient data from a network of GP practices across the UK. Primary care data are linked to a range of other health related data to provide a longitudinal, representative UK population health dataset. The data in CPRD Aurum encompass 40 million research acceptable patients, including 13 million currently registered patients.

Eligible cases and controls will be invited to participate in the study and to report their symptoms, quality of life and work capability monthly for 12 months on a digital platform called Atom5™ produced by the medical technology company Aparito Ltd. Cluster analysis methods will be used to define symptom clusters, which will be further described using data from linked GP records, hospital episode statistics and Office for National Statistics (ONS) mortality records.

A subgroup of participants sampled from each symptom cluster and two matched control groups (those with no history of COVID-19, and those with a history of COVID-19 but who did not develop Long COVID) will be invited to provide blood and saliva samples to measure their immune function, and proteomic and genomic profile (BioWear sub-study). These participants will also be provided a Garmin wearable device to monitor heart rate, oxygen saturation, step count, sleep quality, and post-exertional effects.

## Population and setting

Eligible practices and participants will be identified from the CPRD Aurum database[26] following data extraction using the Data Extraction for Epidemiological Research (DExTER) tool.[27] Practices to enrol in the cohort study will be selected using probability weighted sampling to ensure adequate geographic representation and oversampling of practices with larger numbers of patients with a history of COVID-19, low socioeconomic status, and higher proportions from Black and Ethnic Minority groups, while also preserving the balance of urban versus rural practices. Eligible patients from those practices will be flagged, with oversampling of cases from lower socioeconomic groups, ethnic minorities, and those who received a SARS CoV-2 RT-PCR positive result in 2021.

Participants will need to be actively registered on 31$^{st}$ January 2020 and have at least 12 months of data prior to 31$^{st}$ January 2020. Cases will be defined as those aged 18 years and older with a record of a positive SARS CoV-2 RT-PCR result (index date) and no record of hospitalization within 28 days of the diagnosis. Controls will be defined as individuals without a record of a positive SARS CoV-2 RT-PCR result or suspected or confirmed COVID-19 diagnosis at any time point from 31$^{st}$ January 2020. Each case will be matched to four controls using propensity scores derived from demographic factors (age, sex, ethnic group, and socioeconomic status) and a comprehensive list of comorbidities. Controls will only be eligible if they have not been hospitalized within 28 days of the matched index date.

For peer review only

## Participant recruitment

Participating GP practices will be informed of eligible participants and asked to check their appropriateness for study invitation through the CPRD's Interventional Research Services Platform. Those verified as meeting the eligibility criteria and assessed as appropriate for study invitation by their general practitioner will be sent an invitation letter and patient information sheet, which will provide instructions on how to download the Atom5™ app or access the web portal and a unique onboarding code to register for the study. The study consent form will be hosted on Atom5™, which will include additional eligibility checks to ensure potential participants meet the study criteria. Participants unable to directly access Atom5™, for example due to inadequate technology access, will also be offered a telephone interview to complete the questionnaires with a research nurse. Language translators will also be provided when required.

## Patient reported outcomes (PROs)

Participants enrolled on Atom5™ will be asked to complete a novel electronic patient reported outcome (e-PRO) called the Symptom Burden Questionnaire™ for Long COVID (SBQ-LC™). This includes a comprehensive list of symptoms that were identified from a systematic review of symptoms associated with Long COVID,[5] discussions with Long COVID patients and clinicians, and field testing. Participants will also be asked to complete the Functional Assessment of Chronic Illness Therapy-Fatigue (FACIT-F), Patient Health Questionnaire-2 (PHQ-2), Generalised Anxiety Disorder-2 (GAD-2), Abbreviated PTSD Checklist (PCL-2), modified MRC Dyspnoea Scale,COVID-19 core outcome measure for recovery and Euroqol EQ-5D-5L and answer questions about work capability. Participants will be notified to complete these measures monthly for a total of 12 months.

## Defining symptom clusters

The symptom data captured at baseline in Atom5™ in addition to clinical data from primary care records will be used to define symptom clusters. We will employ clustering algorithms that have been shown to work well in medical settings, with binary and mixed variables: (1) complete linkage hierarchical clustering; (2) probabilistic c-means cluster analysis; and (3) density peaked clustering analysis. We will evaluate algorithm performance and the optimal number of clusters (e.g., by internal validation against a holdout dataset using metrics such as Gap statistic and silhouette index).[28,29]

The demographic and clinical characteristics of individuals in each symptom cluster will be described using data from Atom5™ and linked GP records in CPRD Aurum. This will include age, sex, ethnic group, socioeconomic status, smoking status, body mass index, SARS CoV-2 immunisation status, and comorbidities. Clinical outcomes for each of these clusters will be described using linked Hospital Episode Statistics and ONS mortality data.

## BioWear sub-study

50 participants sampled from the four most prevalent symptom clusters, 50 from the control group without a history of COVID-19, and 50 with a history of COVID-19 but without Long COVID, will be recruited to provide blood and saliva samples. These will be used to measure immune function (inflammatory markers, T-cell function, and autoantibodies), proteomics, and genomic profile. These participants will also be provided a Garmin Vivosmart 4 device to measure heart rate, oxygen saturation,

For peer review only

step count and sleep quality at baseline, 6 months, and 12 months. Notifications will be sent once a month to undertake a 40-step test once a week for each week of device use, to assess for post-exertional effects of Long COVID. Together these data will allow for detailed immunological and digital phenotyping of the newly described symptom clusters.

## Sample size

We aim to recruit 4000 individuals with a history of COVID-19, with a minimum sample of 2000 (Figure 2). We also aim to recruit 1000 matched controls, with a minimum of 500. Reliable detection of clustered data via well-chosen combinations of these methods has been shown to require a minimum of 20-30 observations per subgroup provided good cluster separation exists (effect size Δ=4 or over).[30] We expect that with 500 patients in the Long Covid cohort we will have power above 80% to detect well-separated clusters comprising at least 5% of the cohort for K=4 to K=6 clusters.

## Analysis plan

Unsupervised exploratory clustering techniques will be employed to identify distinct Long COVID symptom clusters. We will first perform dimension reduction using one of: multi-dimensional scaling (MDS), t-stochastic neighbour embedding (t-SNE) and uniform manifold approximation and projection (UMAP). This first step will reduce dimensionality of the global data set while retaining local distances between individuals.

Following the dimensionality reduction step, we will employ clustering algorithms that have been shown to work well in medical settings:

(1) k-means clustering, which locates the stable set of centroids of a predetermined number of k clusters.

(2) Fuzzy c-means cluster analysis, a version of k-means clustering which allows data points to have partial cluster membership.

(3) Hierarchical agglomerative clustering, which joins observations in a recursive fashion satisfying some linkage criterion until k clusters have been generated. We will use the Ward linkage, which joins observations that minimise the variance of merged clusters.

(4) Gaussian finite mixture models, a model-based approach to clustering which associate each data point with a multivariate (ellipsoidal) Gaussian distribution.

(5) DBSCAN/HDBSCAN, algorithms, which identify clusters of dense observations among unassigned lower-density observations.

The optimal number of clusters for all methods (except DBSCAN) will be determined using model selection methods and internal cross validation. Cluster membership goodness of fit will be evaluated by: (1) the Jaccard similarities (intersection size divided by union size) of the original clusters to the most similar clusters in the resampled data, and (2) the average silhouette score, which will vary between +1 for an observation aligned with its cluster centre and -1 for an observation aligned with the centre of another cluster. We will determine mean bootstrapped Jaccard and silhouette indices to assess cluster stability. The plot of within-groups sum of squares vs K will be checked graphically for optimal K (elbow method) and a minimum BIC.

For peer review only

# Other planned work

## Epidemiology of Long COVID in primary care

We will undertake an epidemiological analysis of Long COVID symptoms and clinical outcomes using data from GP records in CPRD Aurum.[31] The prevalence of these symptoms will be compared to those in matched controls. Risk factors associated with the development of long-term symptoms in those with a history of COVID-19 will be described. Cluster analyses will be performed to identify distinct Long COVID phenotypes and their risk of hospital admission and mortality will be assessed.

## Impact of Long COVID on healthcare utilisation and costs

The utilisation of a range of healthcare services (e.g., GP consultations, pharmacotherapy, secondary care referrals, and hospital admissions) within 12 months from the index date will be identified and enumerated for both cases and control groups, with UK unit costs (2021 values) assigned to each component.

To estimate the costs associated with primary care resource use we will measure different types of GP consultations (surgery visits, home visits, and telephone consultations). Unit costs (per visit) for each type of consultation will be derived from the Unit Costs of Health and Social Care (2020).[32] As it is not feasible to cost all medications prescribed for both the cases and control groups during the study period, we will focus only on a broad definition of relevant medications which could include respiratory (e.g., inhaled short acting beta-2 agonists) and cardiovascular (e.g., aspirin) therapies. The costs of prescribed medications will be obtained from NHS Electronic Drug Tariffs, BNF, or Prescription Cost Analysis. Costs associated with hospital admissions will be derived from linked HES Admitted Patient Care (APC) data. Other secondary care costs will be estimated based on secondary care referrals from primary care, which will include outpatient and accident and emergency referrals. The average costs per episode for each type of resource use will be obtained using NHS Reference Costs.[33]

The annual costs of healthcare utilisation for each different area of resource use will then be estimated as a product of the quantities of the resource per year and the attached unit costs. The total costs of all healthcare resource utilisation for each patient will be aggregated to make up the total direct costs of healthcare resource utilisation. Multivariable regression models will be used to estimate the incremental difference in the total all-cause healthcare costs between cases and controls controlling for demographic, clinical, and other confounding factors.

## Impact of Long COVID on work capability and economic losses

To understand the broader impacts of long COVID on individuals and families, it is necessary to analyse work capability and economic losses. We will use lost productivity (absenteeism) as a proxy for indirect costs incurred since that is the most feasible cost component that can be captured in our data. Medically related absenteeism days will be estimated by identifying patients who reported work absence/changes in working hours using questions captured within Atom5™, and their linked sick notes record in CPRD. The associated costs will be calculated by applying average national earnings or based on individual employee wage information where available.

The human capital approach which is the most commonly used method for estimating the indirect costs of illnesses will be adopted.[34,35] This method equates the costs of illnesses to the losses of future

For peer review only

total income that patients could have earned had they remained healthy, revealing the opportunity costs associated with long Covid in this case. Indirect costs incurred during the study period will be compared between the case and control groups using Wilcoxon signed-rank tests. Multivariable regression models will assess incremental cost differences between cases and controls, adjusting for confounding factors.

## Post-viral syndromes: a systematic review of symptoms, health impacts, treatments, and their implications for Long COVID

This systematic review will narratively and quantitatively summarise the evidence on the prevalence of symptoms and health impacts (clinical complications and impacts on quality of life and work capability) associated with previous post-viral syndromes (e.g. post-viral effects of the Middle East Respiratory Syndrome). The review will also summarise the evidence from randomised controlled trials and observational studies on non-pharmacological treatments for post-viral syndromes including Long COVID. A review protocol will be published separately.

## Survey of non-prescribed medication, supplements, remedies, and other therapies for Long COVID

A cross-sectional electronic survey will be undertaken to capture the non-prescribed medicines, supplements, remedies and other therapies used by individuals with long COVID to manage their symptoms, and what influenced their decision making. The aim is to identify self-prescribed therapies that are potentially harmful and pose clinical risks. This survey was suggested by members of the patient and public involvement group who reported their awareness of individuals self-medicating with a range of alternative therapies and highlighted the importance of capturing data on these self-management behaviours.

## Consensus and co-production workshops

A series of expert consensus workshops will be convened to make recommendations on non-pharmacological therapies to be used in each Long COVID syndrome, to be evaluated in clinical trials, and to be recommended against, based on the cumulated evidence from the rapid systematic review and primary findings from WP2. The consensus workshop will follow the James Lind Alliance final priority setting method, based on Nominal Group Technique.[36] In addition, decision making will be guided by the APEASE criteria.[37] Particular consideration will be given to non-pharmacological therapies (e.g., physiotherapy, breathing techniques, sleep hygiene) that can be delivered digitally and at scale.

Subsequently, intervention co-production workshops will be held to determine which of the agreed interventions are suitable for or could be adapted for remote delivery. Person-centred design principles will be applied to co-produce a supportive, remotely delivered intervention, guided by the “building blocks of co-production” framework.[38]

Expert consensus and co-production workshops will be attended by people who have experienced Long COVID and their family members, friends, or carers; healthcare professionals; experts in symptoms and potential treatments relevant to Long COVID; regulators; and policy makers.

For peer review only - http://bmjopen.bmj.com/site/about/guidelines.xhtml

### Feasibility study

A feasibility study will be undertaken on the newly coproduced intervention. This will assess the feasibility of participant recruitment, randomisation of eligible participants to the intervention and control groups, capture of intervention process measures, and collecting outcome and resource measures. The results of the feasibility study will help to determine whether to progress to a fully powered randomised controlled trial.

### De-centralised trial platform

An important longer-term aim of the TLC Study is to develop a decentralised trial platform for evaluating therapies for Long COVID. This platform will be able to flag eligible participants using GP record data in CPRD Aurum, recruit participants using the CPRD Intervention Research Service Platform, obtain e-consent and baseline data on Atom5™, and obtain outcome data from a combination of Atom5™ for PROs, and clinical outcomes from GP records and linked hospital episode statistics and ONS mortality data.

### Evaluation of PPI

Throughout the project, we will co-evaluate patient and public involvement (PPI) activities with the PPI members and obtain their views on how empowered they felt to meaningfully contribute to the study. The Guidance for Reporting Involvement of Patients and the Public (GRIPP) 2 checklist will be used for the reporting and evaluation of PPI for the TLC project.[39] Feedback on PPI activity outcomes will be shared with the PPI group.

## Patient and public involvement

A PPI group comprising of up to 15 individuals has been established following the NIHR Include guidance, which includes diverse ethnic and socioeconomic representation.[40] All study plans have been informed through discussions with the group and through broader discussions with national patient support groups (LongCovidSOS, Long Covid Scotland, Long Covid Wales, and Long Covid Support).  This has significantly contributed to the development of the SBQ-LC™ and informed many aspects of the TLC study more broadly. Research findings will be discussed regularly with the group and revised based on their input accordingly. The PPI group will also inform the development of and contribute to dissemination plans, coauthor publications, and prepare lay friendly materials to disseminate research findings to patients and the public. Group members will be reimbursed for their time and any expenses incurred according to the UK standards for public involvement.[41]

## Ethics and dissemination

Ethical approval for the study was provided by the Solihull Research Ethics Committee (21/WM/0203).

For peer review only - http://bmjopen.bmj.com/site/about/guidelines.xhtml

# Figures

***Figure 1*** *Project overview*

***Figure 2*** *Study flow chart*

For peer review only

# References


1 GOV.UK. Cases in United Kingdom. 2021.https://coronavirus.data.gov.uk/details/cases (accessed 19 Oct 2021).

2 Center for Systems Science and Engineering (CSSE) at John Hopkins University. COVID-19 Map. 2021.https://coronavirus.jhu.edu/map.html (accessed 19 Oct 2021).

3 GOV.UK. Deaths in United Kingdom. 2021.https://coronavirus.data.gov.uk/details/deaths (accessed 19 Oct 2021).

4 GOV.UK. Healthcare in United Kingdom. 2021.https://coronavirus.data.gov.uk/details/healthcare (accessed 19 Oct 2021).

5 Aiyegbusi OL, Hughes SE, Turner G, *et al.* Symptoms, complications and management of long COVID: a review: *J R Soc Med* 2021;:1410768211032850. doi:10.1177/01410768211032850

6 Greenhalgh T, Knight M, A'Court C, *et al.* Management of post-acute covid-19 in primary care. *BMJ* 2020;**370**. doi:10.1136/bmj.m3026

7 National Institute for Health and Care Excellence. COVID-19 rapid guideline: managing the long-term effects of COVID-19. 2020.https://www.nice.org.uk/guidance/NG188 (accessed 19 Oct 2021).

8 Galeotti C, Bayry J. Autoimmune and inflammatory diseases following COVID-19. *Nat Rev Rheumatol* 2020;**16**:413–4. doi:10.1038/s41584-020-0448-7

9 Whitaker M, Elliott J, Chadeau-Hyam M, *et al.* Persistent symptoms following SARS-CoV-2 infection in a random community sample of 508,707 people. *medRxiv* Published Online First: 3 July 2021. doi:10.1101/2021.06.28.21259452

10 GOV.UK. ONS: Short report on Long COVID. 2021.https://www.gov.uk/government/publications/ons-short-report-on-long-covid-22-july-2021 (accessed 19 Oct 2021).

11 National Institute for Health Research. NIHR themed review: Living with Covid19. 2020. doi:10.3310/THEMEDREVIEW_41169

12 Mahase E. Long covid could be four different syndromes, review suggests. BMJ. 2020;**371**:m3981. doi:10.1136/bmj.m3981

13 UK Health Security Agency. COVID-19: long-term health effects - GOV.UK. 2020.https://www.gov.uk/government/publications/covid-19-long-term-health-effects (accessed 19 Oct 2021).

14 Sudre CH, Murray B, Varsavsky T, *et al.* Attributes and predictors of long COVID. *Nat Med* 2021;:1–6. doi:10.1038/s41591-021-01292-y

15 Brodin P. Immune determinants of COVID-19 disease presentation and severity. Nat. Med. 2021;**27**:28–33. doi:10.1038/s41591-020-01202-8

16 British Society for Immunology. Long-term immunological health consequences of COVID-19. 2020. https://www.immunology.org/sites/default/files/BSI_Briefing_Note_August_2020_FINAL.pdf


For peer review only

(accessed 19 Oct 2021).

17 Nehme J, Borghesan M, Mackedenski S, *et al.* Cellular senescence as a potential mediator of COVID-19 severity in the elderly. Aging Cell. 2020;**19**:e13237. doi:10.1111/acel.13237

18 Mondelli V, Pariante CM. What can neuroimmunology teach us about the symptoms of long-COVID? *Oxford Open Immunol* 2021;**2**. doi:10.1093/oxfimm/iqab004

19 Recovery. Welcome — RECOVERY Trial. 2020.https://www.recoverytrial.net/ (accessed 19 Oct 2021).

20 PHOSP. Home - PHOSP-COVID . 2020.https://phosp.org/ (accessed 19 Oct 2021).

21 Maxwell E, Poole R. NIHR themed review: Living with Covid19 - Second review. doi:10.3310/themedreview_45225

22 LongCovidSOS. 2021. https://www.longcovidsos.org/ (accessed 22 Oct 2021).

23 Dalmaijer E, Nord CL, Astle D. Statistical power for cluster analysis. *ArXiv* Published Online First: 2020. doi:arXiv:2003.00381

24 Haroon S, Subramanian A, Gkoutos G, *et al.* Long COVID in non-hospitalised individuals: symptoms, risk factors and syndromes. *CPRD* Published Online First: 2021.https://cprd.com/protocol/long-covid-non-hospitalised-individuals-symptoms-risk-factors-and-syndromes (accessed 19 Oct 2021).

25 NHS. National cost collection for the NHS. 2020.https://www.england.nhs.uk/national-cost-collection/ (accessed 26 Oct 2021).

26 Jo C. Cost-of-illness studies: concepts, scopes, and methods. *Clin Mol Hepatol* 2014;**20**:327. doi:10.3350/CMH.2014.20.4.327

27 Choi H-J, Lee E-W. Methodology of estimating socioeconomic burden of disease using national health insurance (NHI) data. *Eval Heal Serv* Published Online First: 18 December 2019. doi:10.5772/INTECHOPEN.89895

28 James Lind Alliance. Chapter 8: Final priority setting. 2020.https://www.jla.nihr.ac.uk/jla-guidebook/chapter-8/final-priority-setting.htm (accessed 19 Oct 2021).

29 Michie S, Atkins L, West R. *The Behaviour Change Wheel: A Guide To Designing Interventions*. London: : Silverback 2014. http://www.behaviourchangewheel.com/ (accessed 19 Oct 2021).

30 Carter S, Steynor A, Vincent K, *et al.* A manual for co-production in African weather and climate services: Home. Cape Town: 2019. https://futureclimateafrica.org/coproduction-manual/ (accessed 19 Oct 2021).

31 Staniszewska S, Brett J, Simera I, *et al.* GRIPP2 reporting checklists: tools to improve reporting of patient and public involvement in research. *BMJ* 2017;**358**:3453. doi:10.1136/bmj.j3453

32 National Institute for Health Research. NIHR INCLUDE Guidance (General) . 2020.https://www.learningforinvolvement.org.uk/?opportunity=nihr-include-guidance-general (accessed 19 Oct 2021).

33 INVOLVE. Policy on payment of fees and expenses for members of the public actively involved with INVOLVE. 2016. http://www.invo.org.uk/posttypepublication/national-institute-for-health-

(accessed 19 Oct 2021).

For peer review only

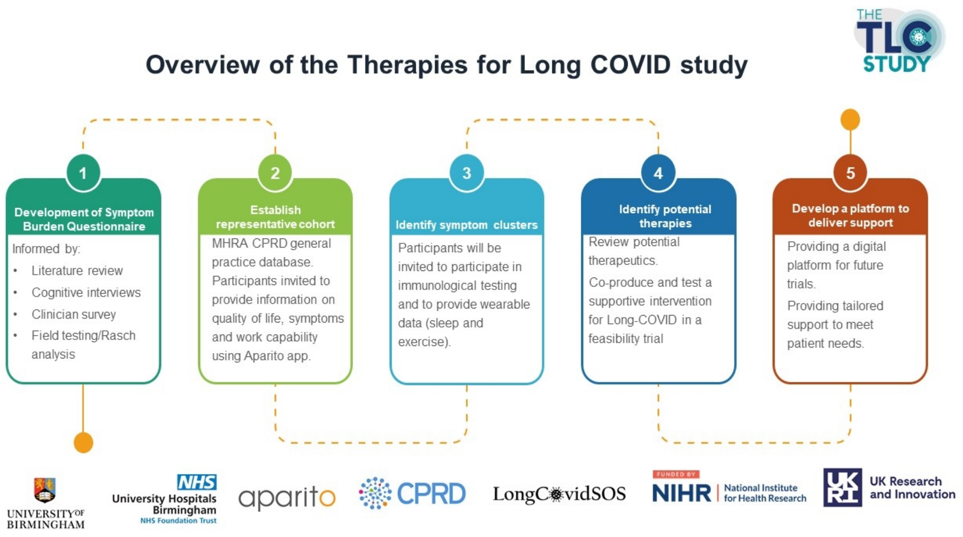


Overview of the Therapies for Long COVID study

338x190mm (96 x 96 DPI)

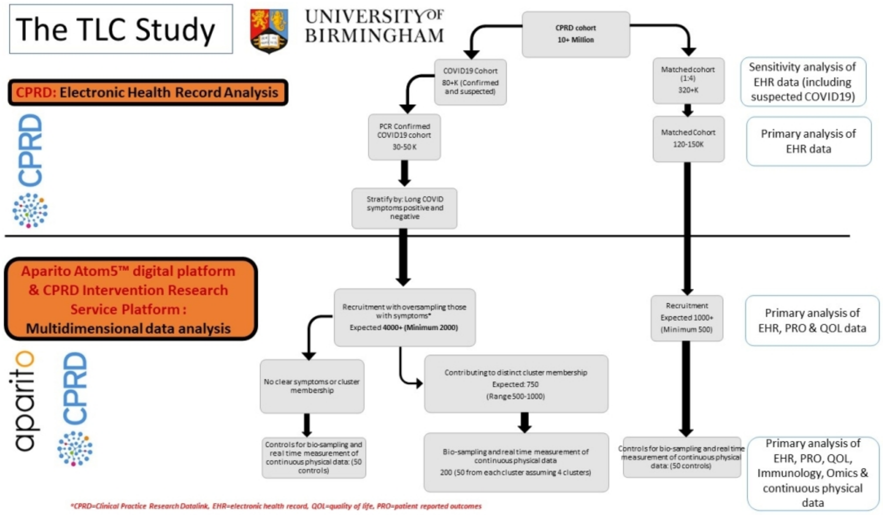


The TLC study

400x250mm (96 x 96 DPI)

BMJ Open

# Therapies for Long COVID in non-hospitalised individuals - from symptoms, patient-reported outcomes, and immunology to targeted therapies (The TLC Study): Study protocol



| | |
|---|---|
| Journal: | *BMJ Open* |
| Manuscript ID | bmjopen-2021-060413.R1 |
| Article Type: | Protocol |
| Date Submitted by the Author: | 22-Mar-2022 |





| | |
|---|---|
| Complete List of Authors: | Haroon, Shamil; University of Birmingham, Institute of Applied Health Research<br>Nirantharakumar, Krishnarajah; University of Birmingham, Institute of Applied Health Research; Midlands Health Data Research UK<br>Hughes, Sarah; University of Birmingham, Institute of Applied Health Research; National Institute for Health Research (NIHR) Applied Research Centre West Midlands<br>Subramanian, Anuradhaa; University of Birmingham, Institute of Applied Health Research<br>Aiyegbusi, Olalekan Lee; University of Birmingham, Institute of Applied Health Research; National Institute for Health Research (NIHR) Applied Research Centre West Midlands<br>Davies, Elin Haf; Aparito Ltd<br>Myles, Puja; Medicines and Healthcare Products Regulatory Agency<br>Williams, Tim; Medicines and Healthcare Products Regulatory Agency<br>Turner, Grace; University of Birmingham, Institute of Applied Health Research<br>Chandan, Joht Singh; University of Birmingham, Institute of Applied Health Research<br>McMullan, Christel; University of Birmingham, Institute of Applied Health Research<br>Lord, Janet; University of Birmingham, Institute of Inflammation and Ageing<br>Wraith, David; University of Birmingham, Institute of Immunology and Immunotherapy<br>McGee, Kirsty; University of Birmingham, Institute of Inflammation and Ageing<br>Denniston, Alastair; University Hospitals Birmingham NHS Foundation Trust<br>Taverner, Thomas ; University of Birmingham, Institute of Applied Health Research<br>Jackson, Louise; University of Birmingham, Institute of Applied Health Research<br>Sapey, Elizabeth; University of Birmingham, Institute of Inflammation and Ageing<br>Gkoutos, George; University of Birmingham, Institute of Cancer and Genomic Sciences<br>Gokhale, Krishna; University of Birmingham, Institute of Applied Health |

| | |
|---|---|
| | Research<br>Leggett, Edward; Medicines and Healthcare Products Regulatory Agency, CPRD<br>Iles, Clare; Medicines and Healthcare Products Regulatory Agency<br>Frost, Christopher; Aparito Ltd<br>McNamara, Gary; Aparito Ltd<br>Bamford, Amy; University Hospitals Birmingham NHS Foundation Trust<br>Marshall, Tom; University of Birmingham, Institute of Applied Health Research<br>Zemedikun, Dawit; University of Birmingham, Institute of Applied Health Research<br>Price, Gary; Not applicable<br>Marwaha, Steven; University of Birmingham, Institute for Mental Health<br>Simms-Williams, Nikita; University of Birmingham, Institute of Applied Health Research<br>Brown, Kirsty; University of Birmingham, Institute of Applied Health Research<br>Walker, Anita; University of Birmingham, Institute of Applied Health Research<br>Jones, Karen; University of Birmingham, Institute of Applied Health Research<br>Matthews, Karen; Not applicable<br>Camaradou, Jennifer; Not applicable<br>Saint-Cricq, Michael; Not applicable<br>Kumar, Sumita; Not applicable<br>Alder, Yvonne; Not applicable<br>Stanton, David E.; Not applicable<br>Agyen, Lisa; Not applicable<br>Baber, Megan; Not applicable<br>Blaize, Hannah; Not applicable<br>Calvert , Melanie ; University of Birmingham, Institute of Applied Health Research; Midlands Health Data Research UK |
| <b>Primary Subject Heading</b>: | Infectious diseases |
| Secondary Subject Heading: | Public health, Infectious diseases, Pharmacology and therapeutics |
| Keywords: | COVID-19, IMMUNOLOGY, PUBLIC HEALTH, THERAPEUTICS |
| | |

# Therapies for Long COVID in non-hospitalised individuals - from symptoms, patient-reported outcomes, and immunology to targeted therapies (The TLC Study): Study protocol

For peer review only

Authors: Shamil Haroon[1] (s.haroon@bham.ac.uk), Krishnarajah Nirantharakumar[1,2] (K.Nirantharan@bham.ac.uk), Sarah E Hughes[1,3,4] (S.E.Hughes@bham.ac.uk), Anuradhaa Subramanian[1] (A.Subramanian@bham.ac.uk), Olalekan Lee Aiyegbusi[1,3,4,5,6] (O.L.Aiyegbusi@bham.ac.uk), Elin Haf Davies[7] (elin@aparito.com), Puja Myles[8] (Puja.Myles@mhra.gov.uk), Tim Williams[8] (Tim.Williams@mhra.gov.uk), Grace M Turner[1,3] (G.Turner.1@bham.ac.uk), Joht Singh Chandan[1] (J.S.Chandan.1@bham.ac.uk), Christel McMullan[1,3] (C.McMullan@bham.ac.uk), Janet Lord[9] (J.M.LORD@bham.ac.uk), David Wraith[10] (D.Wraith@bham.ac.uk), Kirsty McGee[9] (K.C.McGee@bham.ac.uk), Alastair Denniston[11] (A.Denniston@bham.ac.uk), Tom Taverner[1] (T.Taverner@bham.ac.uk), Louise Jackson[1] (L.Jackson.1@bham.ac.uk), Elizabeth Sapey[9] (E.Sapey@bham.ac.uk), Georgios Gkoutos[12] (G.Gkoutos@bham.ac.uk), Krishna Gokhale[1] (K.M.Gokhale@bham.ac.uk), Edward Leggett[8] (Edward.Leggett@mhra.gov.uk), Clare Iles[8] (Clare.Iles@mhra.gov.uk), Christopher Frost[7] (chris.frost@aparito.com), Gary McNamara[7] (gary.mcnamara@aparito.com), Amy Bamford[11] (Amy.Bamford@uhb.nhs.uk), Tom Marshall[1] (T.P.MARSHALL@bham.ac.uk), Dawit Zemedikun[1] (D.T.Zemedikun@bham.ac.uk), Gary Price (tlcstudy@bham.ac.uk), Steven Marwaha[13] (S.Marwaha@bham.ac.uk), Nikita Simms-Williams[1] (N.Simms-Williams@bham.ac.uk), Kirsty Brown[1,14] (BrownKR@adf.bham.ac.uk), Anita Walker[1] (A.A.Walker@bham.ac.uk), Karen Jones[1] (K.Jones.2@bham.ac.uk), Karen Matthews (tlcstudy@bham.ac.uk), Jennifer Camaradou (tlcstudy@bham.ac.uk), Michael Saint-Cricq (tlcstudy@bham.ac.uk), Sumita Kumar (tlcstudy@bham.ac.uk), Yvonne Alder (tlcstudy@bham.ac.uk), David E. Stanton (tlcstudy@bham.ac.uk), Lisa Agyen (tlcstudy@bham.ac.uk), Megan Baber (tlcstudy@bham.ac.uk), Hannah Blaize (tlcstudy@bham.ac.uk), Melanie Calvert[1,2,3,4,5,6] (M.Calvert@bham.ac.uk)

Corresponding author: Shamil Haroon

Institute of Applied Health Research,
College of Medical and Dental Sciences,

University of Birmingham,
Edgbaston,
Birmingham,
B15 2TT
T: +44 (0) 121 414 5042

## Affiliations

1. Institute of Applied Health Research, University of Birmingham, Birmingham, UK
2. Midlands Health Data Research UK, Birmingham, UK
3. Centre for Patient Reported Outcomes Research, Institute of Applied Health Research, University of Birmingham, Birmingham, UK
4. National Institute for Health Research (NIHR) Applied Research Centre West Midlands, Birmingham, UK
5. Birmingham Health Partners Centre for Regulatory Science and Innovation, University of Birmingham, Birmingham, UK
6. NIHR Birmingham Biomedical Research Centre, NIHR Surgical Reconstruction and Microbiology Research Centre, University of Birmingham, Birmingham, UK
7. Aparito Ltd, Wrexham, UK
8. Clinical Practice Research Datalink, Clinical Practice Research Datalink, Medicines and Healthcare Products Regulatory Agency, London, UK
9. Institute of Inflammation and Ageing, University of Birmingham, Birmingham, UK
10. Institute of Immunology and Immunotherapy, University of Birmingham, Birmingham UK
11. University Hospitals Birmingham NHS Foundation Trust, Birmingham, UK
12. Institute of Cancer and Genomic Sciences, University of Birmingham, Birmingham, UK
13. Institute for Mental Health, University of Birmingham, Birmingham, UK
14. School of Sport, Exercise and Rehabilitation Sciences, University of Birmingham, UK

## Author contributions

SH and MC oversaw and contributed to the drafting of the entire manuscript with input from all co-authors. KN, AS, AD, TT, GG provided methodological and statistical expertise. JSC and OLA supported drafting the background and co-led the evidence review on post-viral syndromes. OLA was academic lead for patient and public involvement. KM, GP, JC, MSC, SK, YA, DES, LA, MB and HB provided expert patient input based on their lived experience of COVID and its long-term effects. SEH, MC and CM led the development of the Symptom Burden Questionnaire™ for Long COVID. PM, TW, CI and EL provided expertise on CPRD Aurum data and the Interventional Research Service Platform. EHD and CF provided input on the use of Atom5™, ePROs and wearables. JL and DW provided subject and methodological expertise on the immunological analyses with input from KM. DZ and LJ drafted the sections on health econometrics. GT drafted the section on the over-the-counter medication survey. ES, AB, SM and TM provided clinical and public health expertise. GK supported data extraction from CPRD Aurum and is the creator of DExTER. KB and NSW supported revisions to the manuscript and referencing. AW and KJ provided administrative support for the project. AW assisted with usability testing and protocol development. All co-authors reviewed and approved the manuscript.

For peer review only

## Funding statement

This work is independent research jointly funded by the National Institute for Health Research (NIHR) and UK Research and Innovation (UKRI) (Therapies for Long COVID in non-hospitalised individuals: From symptoms, patient reported outcomes and immunology to targeted therapies (The TLC Study), COV-LT-0013). The views expressed in this publication are those of the author(s) and not necessarily those of the National Institute for Health Research or the Department of Health and Social Care.

## Conflicts of interest

MC is Director of the Birmingham Health Partners Centre for Regulatory Science and Innovation, Director of the Centre for the Centre for Patient Reported Outcomes Research and is a National Institute for Health Research (NIHR) Senior Investigator. MC receives funding from the National Institute for Health Research (NIHR) Birmingham Biomedical Research Centre, the NIHR Surgical Reconstruction and Microbiology Research Centre and NIHR ARC West Midlands at the University of Birmingham and University Hospitals Birmingham NHS Foundation Trust, Health Data Research UK, Innovate UK (part of UK Research and Innovation), Macmillan Cancer Support, UCB Pharma, Janssen, GSK and Gilead. MC has received personal fees from Astellas, Aparito Ltd, CIS Oncology, Takeda, Merck, Daiichi Sankyo, Glaukos, GSK and the Patient-Centered Outcomes Research Institute (PCORI) outside the submitted work.

SEH is supported by the National Institute for Health Research (NIHR) Applied Research Centre (ARC), West Midlands at the University of Birmingham. SEH has received personal fees from Cochlear Ltd and Aparito Ltd outside the submitted work.

OLA receives funding from the NIHR Birmingham Biomedical Research Centre (BRC), NIHR Applied Research Centre (ARC), West Midlands at the University of Birmingham and University Hospitals Birmingham NHS Foundation, Innovate UK (part of UK Research and Innovation), Gilead Sciences Ltd, and Janssen pharmaceuticals, Inc. OLA declares personal fees from Gilead Sciences Ltd, GlaxoSmithKline (GSK) and Merck outside the submitted work.

CM receives funding from the National Institute for Health Research (NIHR) Surgical Reconstruction and Microbiology Research Centre, Innovate UK, and has received personal fees from Aparito Ltd outside the submitted work.

PM, TW, CI and EL are employees of CPRD, the data custodians for CPRD Aurum. CPRD is jointly sponsored by the UK government's Medicines and Healthcare products Regulatory Agency and the National Institute for Health Research (NIHR). As a not-for-profit UK government body, CPRD seeks to recoup the cost of delivering its research services to academic, industry and government researchers through research user license fees.

JC is a lay member on the UK NICE COVID expert panel, a citizen partner to the COVID END Evidence Synthesis Network, PPI lead on the NIHR CICADA ME Study, patient representative at the EAN European Neurology Autonomic Nervous Systems Disorders Working Group, a member of the MRC/UKRI Advanced Pain Discovery Platform, and external board member of Plymouth Institute of Health. She also reports contracts with GSK and Medable.

All other co-authors have no relevant competing interests to declare.

For peer review only

## Data statement

Data for this project are not currently available for access outside the research team. Each of the datasets generated within the project may be shared when finalised but this will require an application to the data controllers. The data may then be released to a specific research team for a specific project dependent on the independent approvals being in place.

## Key words

Long COVID, post-COVID-19 syndrome, symptoms, electronic health records, therapies

## Word count

3961

For peer review only

# Abstract

## Introduction

Individuals with COVID-19 frequently experience symptoms and impaired quality of life beyond 4-12 weeks, commonly referred to as Long COVID. Whether Long COVID is one or several distinct syndromes is unknown. Establishing the evidence base for appropriate therapies is needed. We aim to evaluate the symptom burden and underlying pathophysiology of Long COVID syndromes in non-hospitalised individuals and evaluate potential therapies.

## Methods and analysis

A cohort of 4000 non-hospitalised individuals with a past COVID-19 diagnosis and 1000 matched controls will be selected from anonymised primary care records from the Clinical Practice Research Datalink (CPRD), and invited by their general practitioners to participate on a digital platform (Atom5™). Individuals will report symptoms, quality of life, work capability, and patient reported outcome measures. Data will be collected monthly for one year.

Statistical clustering methods will be used to identify distinct Long COVID symptom clusters. Individuals from the four most prevalent clusters and two control groups will be invited to participate in the BioWear sub-study which will further phenotype Long COVID symptom clusters by measurement of immunological parameters and actigraphy.

We will review existing evidence on interventions for post-viral syndromes and Long COVID to map and prioritise interventions for each newly characterised Long COVID syndrome. Recommendations will be made using the cumulated evidence in an expert consensus workshop. A virtual supportive intervention will be coproduced with patients and health service providers for future evaluation.

Individuals with lived experience of Long COVID will be involved throughout this programme through a patient and public involvement group.

## Ethics and dissemination

Ethical approval was obtained from the Solihull Research Ethics Committee, West Midlands (21/WM/0203). The study is registered on the ISRCTN Registry (1567490). Research findings will be presented at international conferences, in peer-reviewed journals, to Long COVID patient support groups and to policymakers.


For peer review only

# Article Summary

## Strengths and limitations of the study

- The study will generate a nationally representative cohort of individuals with Long COVID recruited from primary care.
- We will recruit controls matched on a wide range of demographic and clinical factors to assess differences in symptoms between people with Long COVID and similar individuals without a history of COVID-19.
- We will use a newly developed electronic patient reported outcome measure (Symptom Burden Questionnaire™) for Long COVID to comprehensively assess a wide range of symptoms highlighted by existing literature, patients, and clinicians.
- Immunological, proteomic, genetic, and wearable data captured in the study will allow deep phenotyping of Long COVID syndromes to help better target therapies.
- A limitation is that a significant proportion of non-hospitalised individuals affected by COVID-19 in the first wave of the pandemic will lack confirmatory testing and will be excluded from recruitment to the study.

For peer review only

# Introduction

Coronavirus Disease 2019 (COVID-19), caused by Severe Acute Respiratory Syndrome Coronavirus 2 (SARS CoV-2), is the most significant pandemic since the Spanish Influenza Pandemic of 1918. There have been over 240 million cases worldwide, including over 8.4 million cases in the UK.[1,2] In the UK, this has resulted in over 550,000 hospital admissions and 160,000 deaths.[3,4]

SARS CoV-2 enters human cells using the angiotensin II receptor, which is widely expressed throughout the body. COVID-19 is consequently a multisystem disorder affecting the lungs, heart, gut, kidneys, brain, and skin. A wide range of symptoms have been associated with COVID-19 including breathlessness, fatigue, fever, myalgia, "brain fog" and anosmia.[5]

Symptoms resolve within 12 weeks in most affected individuals. However, in up to 10% of individuals, symptoms of COVID-19 persist, sometimes in a relapsing remitting manner.[6] This can have significant impacts on physical and neurocognitive functioning, quality of life and work capability.[5] Those having symptoms lasting beyond 12 weeks are referred to as experiencing post-COVID-19 syndrome (NICE 2019) or more widely known as Long COVID.[7]

The cause of Long COVID is poorly understood. However, there is evidence from other post-viral syndromes that may be applicable to Long COVID. Evidence from existing studies on COVID-19 suggest a significant burden of pathophysiological insults and sequelae such as lung scarring, kidney injury, myocarditis and systemic inflammatory states that may promulgate longer term symptoms.[5] It is possible that autoimmune pathways may be triggered by COVID-19, leading to multi-system inflammatory damage.[8]

Current evidence suggests that 2.3-37% of individuals with COVID-19 experience symptoms and impaired quality of life and frequently report experiencing heterogeneous physical and psychological symptoms beyond 12 weeks ("Long COVID") that can be debilitating.[9–11] People living with Long COVID have indicated that they are suffering with a range of symptoms, feel 'abandoned' and 'dismissed' by healthcare providers and receive limited or conflicting advice.[12] The aetiology, pathophysiology and treatments for Long COVID are not well understood, creating an unmet need for the growing number of affected individuals. Although efforts are being made to study Long COVID in hospitalised patients, there is a large unmet need among non-hospitalised individuals. Long COVID may comprise several distinct syndromes yet to be fully characterised.[13]

Existing evidence on Long COVID is based on hospitalised cohorts and non-selected populations that are unlikely to be generalisable to the wider UK population of non-hospitalised individuals. A representative population-based cohort with well-matched controls, ideally derived from primary care, is needed to understand the burden of Long COVID, associated disability and impact on work capability. Current literature suggests the physical and psychological symptoms of Long COVID are highly diverse and may represent several distinct syndromes.[14] The characterisation of these syndromes using real-world data in combination with patient reported outcomes would aid health services to deliver appropriate interventions to meet these health needs.

The aetiology and risk factors for Long COVID also need further investigation as literature suggests a disproportionately higher prevalence of Long COVID among women, older adults and individuals with specific symptom clusters.[11,15,16] This may be due to differing immunological profiles of individuals

For peer review only

who go on to develop Long COVID.[17,18] For example, older adults with COVID-19 show higher levels of senescent T cells.[19] As these are pro-inflammatory, more likely to be autoimmune and tissue-damaging, their persistence could mediate distinct symptoms in Long COVID among these individuals. Understanding the immunological basis of Long COVID would better enable clinicians to offer targeted therapies.[20]

There is an urgent need for evidence-based, accessible interventions, co-produced with patients with lived experience of COVID-19, to better support the growing number of non-hospitalised individuals with Long COVID. We propose to derive treatment recommendations drawing upon existing approaches to the management of post-viral syndromes in addition to a detailed understanding of Long COVID syndromes.

There is also a need to develop a trial platform that can link symptom, primary care, and hospital data with immunological profiling to evaluate interventions for Long COVID in non-hospitalised patients. The Recovery trial and PHOSP-COVID study have demonstrated the strength of platforms where several interventions can be rapidly evaluated in secondary care.[21,22] The PRINCIPLE study was set up for non-hospitalised patients in primary care with acute COVID-19.[23] However, such an infrastructure is still needed for affected individuals with Long COVID in the wider community, and studies such as STIMULATE-ICP are being set up to help address this.[24]

The TLC Study will directly address the patient needs highlighted in the NIHR Evidence Report, by the LongCovidSOS campaign, our patient and public involvement group and patient partners, who have co-designed this study.[13,25] Our research will characterise the symptoms, health impacts and underlying pathophysiology of Long COVID, define its component syndromes in non-hospitalised individuals, and provide resources to support symptom management and nurse-led support for those with the severest symptoms. We will use our findings to co-produce with patients a targeted intervention for Long COVID, tailored to individual patient needs.

For peer review only

## Aims

The aim of the study is to evaluate the symptom burden and underlying pathophysiology of Long COVID syndromes in non-hospitalised individuals and the impact on quality of life and work capability. We also aim to identify potential therapies and co-produce a remotely delivered intervention for supporting individuals with Long COVID syndromes. These findings will be used to inform a feasibility study to evaluate the intervention produced during this project, and to plan future evaluations of supportive and pharmacological interventions for treating Long COVID. The methods for therapy identification, intervention development and feasibility testing will be reported in separate linked protocols.

The key objectives are:

1. To establish a representative population-based cohort of non-hospitalised individuals with COVID-19 and prolonged symptoms ("Long COVID").
2. To validate a new patient-reported outcome (PRO) measure of symptom burden in individuals with Long COVID- the Symptom Burden Questionnaire ™ for Long COVID.
3. To collect validated PROs including symptoms, quality of life and work capability data longitudinally from the representative cohort of non-hospitalised individuals with Long COVID.
4. To identify distinct symptom clusters ("syndromes") in individuals with Long COVID and compare these Long COVID syndromes to known post-viral syndromes.
5. To measure immunological, proteomic and miRNA biomarkers to determine the underlying pathogenesis associated with Long COVID syndromes. To capture physical health measures via a wearable device to determine the physical exertions (heart rate intensity and movement) and sleep patterns observed in Long COVID syndromes.
6. To make recommendations on pharmacological and supportive therapies that: i) should be implemented without need for further evaluation; ii) should be evaluated in a clinical trial, and iii) should not be recommended for treating long COVID based on the available evidence.
7. Co-produce a supportive, remotely delivered intervention for individuals with Long COVID, to address the symptoms associated with each Long COVID syndrome.
8. To evaluate patient and public involvement in the study.

For peer review only

# Methods and analysis

## Study design

We will undertake a population-based cohort study of non-hospitalised individuals with Long COVID (cases) and matched controls recruited from general practices in England that contribute data to the Clinical Practice Research Datalink (CPRD) Aurum database (Figure 1).[26] CPRD is a real-world research service supporting retrospective and prospective public health and clinical studies. CPRD is jointly sponsored by the Medicines and Healthcare products Regulatory Agency and the National Institute for Health Research (NIHR), as part of the Department of Health and Social Care. CPRD collects anonymised patient data from a network of GP practices across the UK. Primary care data are linked to a range of other health related data to provide a longitudinal, representative UK population health dataset. The data in CPRD Aurum encompass 40 million research acceptable patients, including 13 million currently registered patients.

Eligible cases and controls will be invited to participate in the study and to report their symptoms, quality of life and work capability monthly for 12 months on a digital platform called Atom5™ produced by the medical technology company Aparito Ltd. Cluster analysis methods will be used to define symptom clusters, which will be further described using data from linked GP records, hospital episode statistics and Office for National Statistics (ONS) mortality records.

A subgroup of participants sampled from each symptom cluster and two matched control groups (those with no history of COVID-19, and those with a history of COVID-19 but who did not develop Long COVID) will be invited to provide blood and saliva samples to measure their immune function, and proteomic and genomic profile (BioWear sub-study). These participants will also be provided a Garmin wearable device to monitor heart rate, oxygen saturation, step count, sleep quality, and post-exertional effects. The start and end dates for the project will be March 2021 to February 2022. Recruitment will take place from November 2021 to May 2022.

## Population and setting

Eligible practices and participants will be identified from the CPRD Aurum database[26] following data extraction using the Data Extraction for Epidemiological Research (DExTER) tool.[27] Practices to enrol in the cohort study will be selected using probability weighted sampling to ensure adequate geographic representation and oversampling of practices with larger numbers of patients with a history of COVID-19, low socioeconomic status, and higher proportions from Black and Ethnic Minority groups, while also preserving the balance of urban versus rural practices. Eligible patients from those practices will be flagged, with oversampling of cases from lower socioeconomic groups, ethnic minorities, and those who received a SARS CoV-2 RT-PCR positive result in 2021.

Participants will need to be actively registered on 31st January 2020 and have at least 12 months of data prior to 31st January 2020. Cases will be defined as those aged 18 years and older with a record of a positive SARS CoV-2 RT-PCR result (index date) and no record of hospitalization within 28 days of the diagnosis. Controls will be defined as individuals without a record of a positive SARS CoV-2 RT-PCR result or suspected or confirmed COVID-19 diagnosis at any time point from 31st January 2020. Each case will be matched to four controls using propensity scores derived from demographic factors (age, sex, ethnic group, and socioeconomic status) and a comprehensive list of comorbidities. Controls will only be eligible if they have not been hospitalized within 28 days of the matched index date.

## Participant recruitment

Participating GP practices will be informed of eligible participants and asked to check their appropriateness for study invitation through the CPRD's Interventional Research Services Platform. Those verified as meeting the eligibility criteria and assessed as appropriate for study invitation by their general practitioner will be sent an invitation letter and patient information sheet, which will provide instructions on how to download the Atom5™ app or access the web portal and a unique onboarding code to register for the study. The study consent form will be hosted on Atom5™, which will include additional eligibility checks to ensure potential participants meet the study criteria. Participants unable to directly access Atom5™, for example due to inadequate technology access, will also be offered a telephone interview to complete the questionnaires with a research nurse. Language translators will also be provided when required.

## Patient reported outcomes (PROs)

Participants enrolled on Atom5™ will be asked to complete a novel electronic patient reported outcome (e-PRO) called the Symptom Burden Questionnaire™ for Long COVID (SBQ-LC™). This includes a comprehensive list of symptoms that were identified from a systematic review of symptoms associated with Long COVID,[5] discussions with Long COVID patients and clinicians, and field testing. Participants will also be asked to complete the Functional Assessment of Chronic Illness Therapy-Fatigue (FACIT-F), Patient Health Questionnaire-2 (PHQ-2), Generalised Anxiety Disorder-2 (GAD-2), Abbreviated PTSD Checklist (PCL-2), modified MRC Dyspnoea Scale,COVID-19 core outcome measure for recovery and Euroqol EQ-5D-5L and answer questions about work capability. Participants will be notified to complete these measures monthly for a total of 12 months.

## Defining symptom clusters

The symptom data captured at baseline in Atom5™ in addition to clinical data from primary care records will be used to define symptom clusters. We will employ clustering algorithms that have been shown to work well in medical settings, with binary and mixed variables: (1) complete linkage hierarchical clustering; (2) probabilistic c-means cluster analysis; and (3) density peaked clustering analysis. We will evaluate algorithm performance and the optimal number of clusters (e.g., by internal validation against a holdout dataset using metrics such as Gap statistic and silhouette index).[28,29]

The demographic and clinical characteristics of individuals in each symptom cluster will be described using data from Atom5™ and linked GP records in CPRD Aurum. This will include age, sex, ethnic group, socioeconomic status, smoking status, body mass index, SARS CoV-2 immunisation status, and comorbidities. Clinical outcomes for each of these clusters will be described using linked Hospital Episode Statistics and ONS mortality data.

## BioWear sub-study

50 participants sampled from the four most prevalent symptom clusters, 50 from the control group without a history of COVID-19, and 50 with a history of COVID-19 but without Long COVID, will be recruited to provide blood and saliva samples at the Clinical Research Facility at University Hospitals Birmingham or alternatively at the participant's home. These samples will be used to measure immune function (inflammatory markers, T-cell function, and autoantibodies), proteomics, and genomic profile.

For peer review only

We will analyse a broad spectrum of anti-neutrophil and organ-specific autoantibodies in serum samples by indirect immunofluorescence including but not restricted to adrenal, autoimmune encephalitis, anti-neutrophil (ANA), anti-neutrophil cytoplasmic antibodies (ANCA), cardiac, epidermal, islet cell, a range of cerebellar (Purkinje cell) antibodies, smooth muscle, mitochondrial, gastric parietal cell, skeletal muscle and endomysial antibodies. The assays undertaken included the full range of autoimmune tests available in an accredited ISO 15189:2012 National Health Service (NHS) Clinical Immunology laboratory. For the plasma proteomic study, we will use a broad platform, Somascan, which detects 7000 proteins. This will include 51 inflammatory proteins that will allow us to estimate the degree of immune ageing in different Long COVID clusters. The additional data will include biomarkers previously shown to be associated with an increased risk of a range of diseases including heart disease, respiratory disease and frailty, allowing us insight into the long-term outcomes of our volunteers."

These participants will also be provided a Garmin Vivosmart 4 device to measure heart rate, oxygen saturation, step count and sleep quality at baseline, 6 months, and 12 months. Notifications will be sent once a month to undertake a 40-step test once a week for each week of device use, to assess for post-exertional effects of Long COVID. Together these data will allow for detailed immunological and digital phenotyping of the newly described symptom clusters.

## Sample size

We aim to recruit 4000 individuals with a history of COVID-19, with a minimum sample of 2000 (Figure 2). We also aim to recruit 1000 matched controls, with a minimum of 500. Reliable detection of clustered data via well-chosen combinations of these methods has been shown to require a minimum of 20-30 observations per subgroup provided good cluster separation exists (effect size $\Delta$=4 or over).[30] We expect that with 500 patients in the Long Covid cohort we will have power above 80% to detect well-separated clusters comprising at least 5% of the cohort for K=4 to K=6 clusters.

## Analysis plan

Unsupervised exploratory clustering techniques will be employed to identify distinct Long COVID symptom clusters. We will first perform dimension reduction using one of: multi-dimensional scaling (MDS), t-stochastic neighbour embedding (t-SNE) and uniform manifold approximation and projection (UMAP). This first step will reduce dimensionality of the global data set while retaining local distances between individuals.

Following the dimensionality reduction step, we will employ clustering algorithms that have been shown to work well in medical settings:

(1) k-means clustering, which locates the stable set of centroids of a predetermined number of k clusters.

(2) Fuzzy c-means cluster analysis, a version of k-means clustering which allows data points to have partial cluster membership.

(3) Hierarchical agglomerative clustering, which joins observations in a recursive fashion satisfying some linkage criterion until k clusters have been generated. We will use the Ward linkage, which joins observations that minimise the variance of merged clusters.

For peer review only

(4) Gaussian finite mixture models, a model-based approach to clustering which associate each data point with a multivariate (ellipsoidal) Gaussian distribution.

(5) DBSCAN/HDBSCAN, algorithms, which identify clusters of dense observations among unassigned lower-density observations.

The optimal number of clusters for all methods (except DBSCAN) will be determined using model selection methods and internal cross validation. Cluster membership goodness of fit will be evaluated by: (1) the Jaccard similarities (intersection size divided by union size) of the original clusters to the most similar clusters in the resampled data, and (2) the average silhouette score, which will vary between +1 for an observation aligned with its cluster centre and -1 for an observation aligned with the centre of another cluster. We will determine mean bootstrapped Jaccard and silhouette indices to assess cluster stability. The plot of within-groups sum of squares vs K will be checked graphically for optimal K (elbow method) and a minimum BIC.

## Other planned work

### Epidemiology of Long COVID in primary care

We will undertake an epidemiological analysis of Long COVID symptoms and clinical outcomes using data from GP records in CPRD Aurum.[31] The prevalence of these symptoms will be compared to those in matched controls. Risk factors associated with the development of long-term symptoms in those with a history of COVID-19 will be described. Cluster analyses will be performed to identify distinct Long COVID phenotypes and their risk of hospital admission and mortality will be assessed.

### Impact of Long COVID on healthcare utilisation and costs

The utilisation of a range of healthcare services (e.g., GP consultations, pharmacotherapy, secondary care referrals, and hospital admissions) within 12 months from the index date will be identified and enumerated for both cases and control groups, with UK unit costs (2021 values) assigned to each component.

To estimate the costs associated with primary care resource use we will measure different types of GP consultations (surgery visits, home visits, and telephone consultations). Unit costs (per visit) for each type of consultation will be derived from the Unit Costs of Health and Social Care (2020).[32] As it is not feasible to cost all medications prescribed for both the cases and control groups during the study period, we will focus only on a broad definition of relevant medications which could include respiratory (e.g., inhaled short acting beta-2 agonists) and cardiovascular (e.g., aspirin) therapies. The costs of prescribed medications will be obtained from NHS Electronic Drug Tariffs, BNF, or Prescription Cost Analysis. Costs associated with hospital admissions will be derived from linked HES Admitted Patient Care (APC) data. Other secondary care costs will be estimated based on secondary care referrals from primary care, which will include outpatient and accident and emergency referrals. The average costs per episode for each type of resource use will be obtained using NHS Reference Costs.[33]

The annual costs of healthcare utilisation for each different area of resource use will then be estimated as a product of the quantities of the resource per year and the attached unit costs. The total costs of all healthcare resource utilisation for each patient will be aggregated to make up the total direct costs of healthcare resource utilisation. Multivariable regression models will be used to estimate the

For peer review only

incremental difference in the total all-cause healthcare costs between cases and controls controlling for demographic, clinical, and other confounding factors.

## Impact of Long COVID on work capability and economic losses

To understand the broader impacts of long COVID on individuals and families, it is necessary to analyse work capability and economic losses. We will use lost productivity (absenteeism) as a proxy for indirect costs incurred since that is the most feasible cost component that can be captured in our data. Medically related absenteeism days will be estimated by identifying patients who reported work absence/changes in working hours using questions captured within Atom5™, and their linked sick notes record in CPRD. The associated costs will be calculated by applying average national earnings or based on individual employee wage information where available.

The human capital approach which is the most commonly used method for estimating the indirect costs of illnesses will be adopted.[34,35] This method equates the costs of illnesses to the losses of future total income that patients could have earned had they remained healthy, revealing the opportunity costs associated with long Covid in this case. Indirect costs incurred during the study period will be compared between the case and control groups using Wilcoxon signed-rank tests. Multivariable regression models will assess incremental cost differences between cases and controls, adjusting for confounding factors.

## Post-viral syndromes: a systematic review of symptoms, health impacts, treatments, and their implications for Long COVID

This systematic review will narratively and quantitatively summarise the evidence on the prevalence of symptoms and health impacts (clinical complications and impacts on quality of life and work capability) associated with previous post-viral syndromes (e.g. post-viral effects of the Middle East Respiratory Syndrome). The review will also summarise the evidence from randomised controlled trials and observational studies on non-pharmacological treatments for post-viral syndromes including Long COVID. A review protocol will be published separately.

## Survey of non-prescribed medication, supplements, remedies, and other therapies for Long COVID

A cross-sectional electronic survey will be undertaken to capture the non-prescribed medicines, supplements, remedies and other therapies used by individuals with long COVID to manage their symptoms, and what influenced their decision making. The aim is to identify self-prescribed therapies that are potentially harmful and pose clinical risks. This survey was suggested by members of the patient and public involvement group who reported their awareness of individuals self-medicating with a range of alternative therapies and highlighted the importance of capturing data on these self-management behaviours.

## Consensus and co-production workshops

A series of expert consensus workshops will be convened to make recommendations on non-pharmacological therapies to be evaluated in clinical trials, and to be recommended against, based on the cumulated evidence from the rapid systematic review and primary findings from WP2. The

For peer review only

consensus workshop will follow the James Lind Alliance final priority setting method, based on Nominal Group Technique.[36] In addition, decision making will be guided by the APEASE criteria.[37] Particular consideration will be given to non-pharmacological therapies (e.g., physiotherapy, breathing techniques, sleep hygiene) that can be delivered digitally and at scale.

Subsequently, intervention co-production workshops will be held to determine which of the agreed interventions are suitable for or could be adapted for remote delivery. Person-centred design principles will be applied to co-produce a supportive, remotely delivered intervention, or identify and select existing tools or resources for evaluation via Atom5™, guided by the "building blocks of co-production" framework.[38]

Expert consensus and co-production workshops will be attended by people who have experienced Long COVID and their family members, friends, or carers; healthcare professionals; experts in symptoms and potential treatments relevant to Long COVID; regulators; and policy makers.

### Feasibility study

A feasibility study will be undertaken on the newly coproduced intervention. This will assess the feasibility of participant recruitment, randomisation of eligible participants to the intervention and control groups, capture of intervention process measures, and collecting outcome and resource measures. The results of the feasibility study will help to determine whether to progress to a fully powered randomised controlled trial.

### De-centralised trial platform

An important longer-term aim of the TLC Study is to develop a decentralised trial platform for evaluating therapies for Long COVID. This platform will be able to flag eligible participants using GP record data in CPRD Aurum, recruit participants using the CPRD Intervention Research Service Platform, obtain e-consent and baseline data on Atom5™, and obtain outcome data from a combination of Atom5™ for PROs, and clinical outcomes from GP records and linked hospital episode statistics and ONS mortality data.

### Evaluation of PPI

Throughout the project, we will co-evaluate patient and public involvement (PPI) activities with the PPI members and obtain their views on how empowered they felt to meaningfully contribute to the study. The Guidance for Reporting Involvement of Patients and the Public (GRIPP) 2 checklist will be used for the reporting and evaluation of PPI for the TLC project.[39] Feedback on PPI activity outcomes will be shared with the PPI group.

## Patient and public involvement

A PPI group comprising of up to 15 individuals has been established following the NIHR Include guidance, which includes diverse ethnic and socioeconomic representation.[40] All study plans have been informed through discussions with the group and through broader discussions with national patient support groups (LongCovidSOS, Long Covid Scotland, Long Covid Wales, and Long Covid Support). This has significantly contributed to the development of the SBQ-LC™ and informed many aspects of the TLC study more broadly. Research findings will be discussed regularly with the group and

revised based on their input accordingly. The PPI group will also inform the development of and contribute to dissemination plans, coauthor publications, and prepare lay friendly materials to disseminate research findings to patients and the public. Group members will be reimbursed for their time and any expenses incurred according to the UK standards for public involvement.[41]

## Ethics and dissemination

Ethical approval for the study was provided by the Solihull Research Ethics Committee (21/WM/0203).

For peer review only

# Figures

***Figure 1*** *Project overview*

***Figure 2*** *Study flow chart*

For peer review only

# References


1 GOV.UK. Cases in United Kingdom. 2021.https://coronavirus.data.gov.uk/details/cases (accessed 19 Oct 2021).

2 Center for Systems Science and Engineering (CSSE) at John Hopkins University. COVID-19 Map. 2021.https://coronavirus.jhu.edu/map.html (accessed 19 Oct 2021).

3 GOV.UK. Deaths in United Kingdom. 2021.https://coronavirus.data.gov.uk/details/deaths (accessed 19 Oct 2021).

4 GOV.UK. Healthcare in United Kingdom. 2021.https://coronavirus.data.gov.uk/details/healthcare (accessed 19 Oct 2021).

5 Aiyegbusi OL, Hughes SE, Turner G, *et al.* Symptoms, complications and management of long COVID: a review: *J R Soc Med* 2021;:1410768211032850. doi:10.1177/01410768211032850

6 Greenhalgh T, Knight M, A'Court C, *et al.* Management of post-acute covid-19 in primary care. *BMJ* 2020;**370**. doi:10.1136/bmj.m3026

7 National Institute for Health and Care Excellence. COVID-19 rapid guideline: managing the long-term effects of COVID-19. 2020.https://www.nice.org.uk/guidance/NG188 (accessed 19 Oct 2021).

8 Galeotti C, Bayry J. Autoimmune and inflammatory diseases following COVID-19. *Nat Rev Rheumatol* 2020;**16**:413–4. doi:10.1038/s41584-020-0448-7

9 Whitaker M, Elliott J, Chadeau-Hyam M, *et al.* Persistent symptoms following SARS-CoV-2 infection in a random community sample of 508,707 people. *medRxiv* Published Online First: 3 July 2021. doi:10.1101/2021.06.28.21259452

10 Office for National Statistics. Prevalence of ongoing symptoms following coronavirus (COVID-19) infection in the UK: 1 April 2021. 2021. https://www.ons.gov.uk/peoplepopulationandcommunity/healthandsocialcare/conditionsanddiseases/bulletins/prevalenceofongoingsymptomsfollowingcoronaviruscovid19infectionintheuk/1april2021 (accessed 28 Oct 2021).

11 Sudre CH, Murray B, Varsavsky T, *et al.* Attributes and predictors of long COVID. *Nat Med* 2021;:1–6. doi:10.1038/s41591-021-01292-y

12 National Institute for Health Research. NIHR themed review: Living with Covid19. 2020. doi:10.3310/THEMEDREVIEW_41169

13 Maxwell E, Poole R. NIHR themed review: Living with Covid19 - Second review. doi:10.3310/themedreview_45225

14 Mahase E. Long covid could be four different syndromes, review suggests. BMJ. 2020;**371**:m3981. doi:10.1136/bmj.m3981

15 Nabavi N. Long covid: How to define it and how to manage it. BMJ. 2020;**370**. doi:10.1136/BMJ.M3489

16 CH S, KA L, MN L, *et al.* Symptom clusters in COVID-19: A potential clinical prediction tool from the COVID Symptom Study app. *Sci Adv* 2021;**7**. doi:10.1126/SCIADV.ABD4177


For peer review only

17 Brodin P. Immune determinants of COVID-19 disease presentation and severity. Nat. Med. 2021;**27**:28–33. doi:10.1038/s41591-020-01202-8

18 British Society for Immunology. Long-term immunological health consequences of COVID-19. 2020. https://www.immunology.org/sites/default/files/BSI_Briefing_Note_August_2020_FINAL.pdf (accessed 19 Oct 2021).

19 Nehme J, Borghesan M, Mackedenski S, *et al.* Cellular senescence as a potential mediator of COVID-19 severity in the elderly. Aging Cell. 2020;**19**:e13237. doi:10.1111/acel.13237

20 Mondelli V, Pariante CM. What can neuroimmunology teach us about the symptoms of long-COVID? *Oxford Open Immunol* 2021;**2**. doi:10.1093/oxfimm/iqab004

21 Recovery. Welcome — RECOVERY Trial. 2020.https://www.recoverytrial.net/ (accessed 19 Oct 2021).

22 PHOSP. Home - PHOSP-COVID . 2020.https://phosp.org/ (accessed 19 Oct 2021).

23 PRINCIPLE. Help find treatments for COVID-19 from home. 2021.https://www.principletrial.org/ (accessed 2 Nov 2021).

24 NIHR. STIMULATE-ICP: Understanding long COVID to improve diagnosis, treatment and care. 2020.https://www.arc-nt.nihr.ac.uk/research/projects/stimulate-icp-improving-diagnosis-treatment-and-care-of-long-covid/ (accessed 2 Nov 2021).

25 LongCovidSOS. 2021. https://www.longcovidsos.org/ (accessed 22 Oct 2021).

26 Wolf A, Dedman D, Campbell J, *et al.* Data resource profile: Clinical Practice Research Datalink (CPRD) Aurum. *Int J Epidemiol* 2019;**48**:1740-1740g. doi:10.1093/IJE/DYZ034

27 Gokhale KM, Chandan JS, Toulis K, *et al.* Data extraction for epidemiological research (DExtER): a novel tool for automated clinical epidemiology studies. *Eur J Epidemiol* 2021;**36**:165–78. doi:10.1007/S10654-020-00677-6/TABLES/6

28 Tibshirani R, Walther G, Hastie T. Estimating the number of clusters in a data set via the gap statistic. *J R Stat Soc Ser B (Statistical Methodol* 2001;**63**:411–23. doi:10.1111/1467-9868.00293

29 Rousseeuw PJ. Silhouettes: A graphical aid to the interpretation and validation of cluster analysis. *J Comput Appl Math* 1987;**20**:53–65. doi:10.1016/0377-0427(87)90125-7

30 Dalmaijer E, Nord CL, Astle D. Statistical power for cluster analysis. *ArXiv* Published Online First: 2020. doi:arXiv:2003.00381

31 Haroon S, Subramanian A, Gkoutos G, *et al.* Long COVID in non-hospitalised individuals: symptoms, risk factors and syndromes. *CPRD* Published Online First: 2021.https://cprd.com/protocol/long-covid-non-hospitalised-individuals-symptoms-risk-factors-and-syndromes (accessed 19 Oct 2021).

32 Curtis L, Burns A. Unit Costs of Health and Social Care 2020, Personal Social Services Research Unit. Canterbury: 2020. doi:10.22024/UniKent/01.02.84818

33 NHS. National cost collection for the NHS. 2020.https://www.england.nhs.uk/national-cost-collection/ (accessed 26 Oct 2021).

For peer review only

34 Jo C. Cost-of-illness studies: concepts, scopes, and methods. *Clin Mol Hepatol* 2014;**20**:327. doi:10.3350/CMH.2014.20.4.327

35 Choi H-J, Lee E-W. Methodology of estimating socioeconomic burden of disease using national health insurance (NHI) data. *Eval Heal Serv* Published Online First: 18 December 2019. doi:10.5772/INTECHOPEN.89895

36 James Lind Alliance. Chapter 8: Final priority setting. 2020.https://www.jla.nihr.ac.uk/jla-guidebook/chapter-8/final-priority-setting.htm (accessed 19 Oct 2021).

37 Michie S, Atkins L, West R. *The Behaviour Change Wheel: A Guide To Designing Interventions*. London: : Silverback 2014. http://www.behaviourchangewheel.com/ (accessed 19 Oct 2021).

38 Carter S, Steynor A, Vincent K, *et al.* A manual for co-production in African weather and climate services: Home. Cape Town: 2019. https://futureclimateafrica.org/coproduction-manual/ (accessed 19 Oct 2021).

39 Staniszewska S, Brett J, Simera I, *et al.* GRIPP2 reporting checklists: tools to improve reporting of patient and public involvement in research. *BMJ* 2017;**358**:3453. doi:10.1136/bmj.j3453

40 National Institute for Health Research. NIHR INCLUDE Guidance (General) . 2020.https://www.learningforinvolvement.org.uk/?opportunity=nihr-include-guidance-general (accessed 19 Oct 2021).

41 National Institute for Health Research, Chief Scientist Office, Health and Care Research Wales, *et al.* UK standards for public Involvement. 2019.https://sites.google.com/nihr.ac.uk/pi-standards/standards?authuser=0 (accessed 2 Nov 2021).

For peer review only

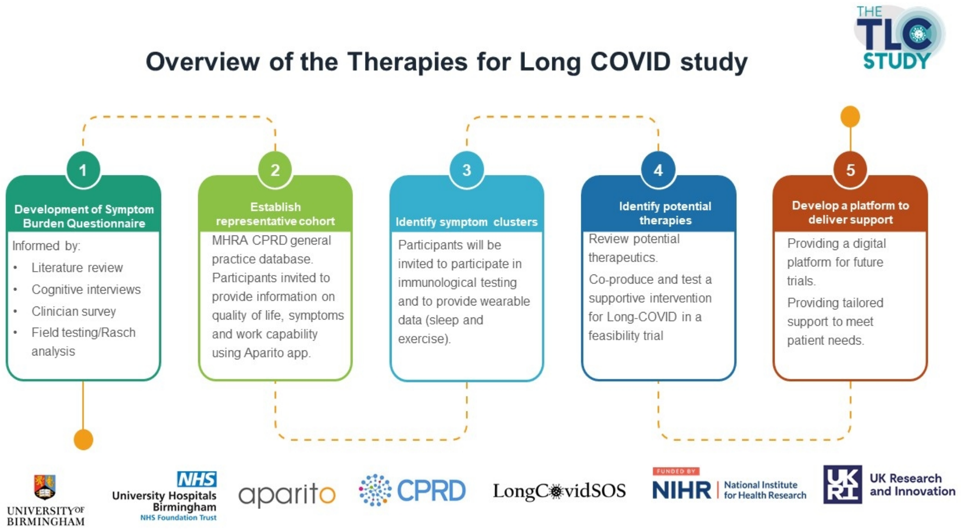


Overview of the Therapies for Long COVID study

338x190mm (96 x 96 DPI)

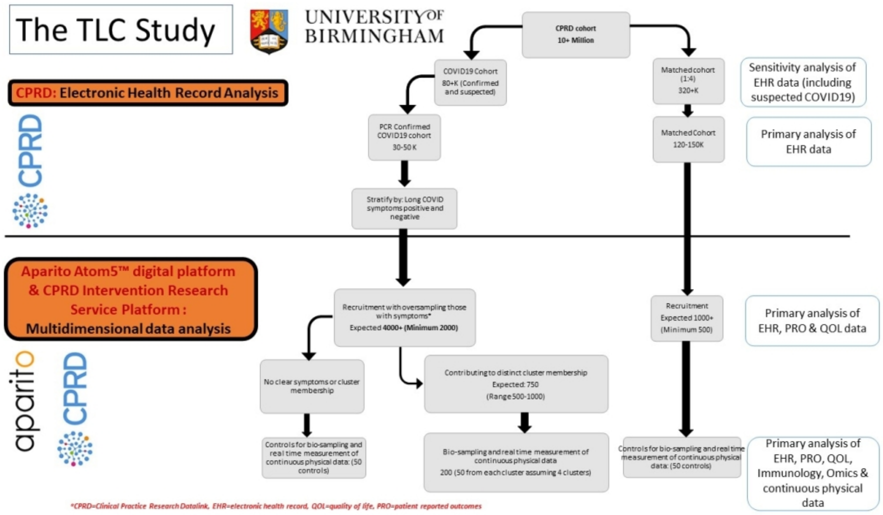


The TLC study

400x250mm (96 x 96 DPI)

STROBE Statement—checklist of items that should be included in reports of observational studies

| | Item No | Recommendation | Page No |
|---|---|---|---|
| **Title and abstract** | 1 | (*a*) Indicate the study's design with a commonly used term in the title or the abstract | 5 |
| | | (*b*) Provide in the abstract an informative and balanced summary of what was done and what was found | 5 |
| **Introduction** | | | |
| Background/rationale | 2 | Explain the scientific background and rationale for the investigation being reported | 7,8 |
| Objectives | 3 | State specific objectives, including any prespecified hypotheses | 9 |
| **Methods** | | | |
| Study design | 4 | Present key elements of study design early in the paper | 10 |
| Setting | 5 | Describe the setting, locations, and relevant dates, including periods of recruitment, exposure, follow-up, and data collection | 10 |
| Participants | 6 | (*a*) *Cohort study*—Give the eligibility criteria, and the sources and methods of selection of participants. Describe methods of follow-up<br>*Case-control study*—Give the eligibility criteria, and the sources and methods of case ascertainment and control selection. Give the rationale for the choice of cases and controls<br>*Cross-sectional study*—Give the eligibility criteria, and the sources and methods of selection of participants | 10,11 |
| | | (*b*) *Cohort study*—For matched studies, give matching criteria and number of exposed and unexposed<br>*Case-control study*—For matched studies, give matching criteria and the number of controls per case | |
| Variables | 7 | Clearly define all outcomes, exposures, predictors, potential confounders, and effect modifiers. Give diagnostic criteria, if applicable | 11 |
| Data sources/ measurement | 8* | For each variable of interest, give sources of data and details of methods of assessment (measurement). Describe comparability of assessment methods if there is more than one group | 9, 11 |
| Bias | 9 | Describe any efforts to address potential sources of bias | 11 |
| Study size | 10 | Explain how the study size was arrived at | 12 |
| Quantitative variables | 11 | Explain how quantitative variables were handled in the analyses. If applicable, describe which groupings were chosen and why | N/A |
| Statistical methods | 12 | (*a*) Describe all statistical methods, including those used to control for confounding | 12 |
| | | (*b*) Describe any methods used to examine subgroups and interactions | N/A |
| | | (*c*) Explain how missing data were addressed | N/A |
| | | (*d*) *Cohort study*—If applicable, explain how loss to follow-up was addressed<br>*Case-control study*—If applicable, explain how matching of cases and controls was addressed<br>*Cross-sectional study*—If applicable, describe analytical methods taking account of sampling strategy | N/A |
| | | (*e*) Describe any sensitivity analyses | N/A |

Continued on next page



For peer review only - http://bmjopen.bmj.com/site/about/guidelines.xhtml

| | | | |
|---|---|---|---|
| **Results** | | | |
| Participants | 13* | (a) Report numbers of individuals at each stage of study—eg numbers potentially eligible, examined for eligibility, confirmed eligible, included in the study, completing follow-up, and analysed | N/A |
| | | (b) Give reasons for non-participation at each stage | N/A |
| | | (c) Consider use of a flow diagram | N/A |
| Descriptive data | 14* | (a) Give characteristics of study participants (eg demographic, clinical, social) and information on exposures and potential confounders | N/A |
| | | (b) Indicate number of participants with missing data for each variable of interest | N/A |
| | | (c) *Cohort study*—Summarise follow-up time (eg, average and total amount) | N/A |
| Outcome data | 15* | *Cohort study*—Report numbers of outcome events or summary measures over time | N/A |
| | | *Case-control study*—Report numbers in each exposure category, or summary measures of exposure | N/A |
| | | *Cross-sectional study*—Report numbers of outcome events or summary measures | N/A |
| Main results | 16 | (*a*) Give unadjusted estimates and, if applicable, confounder-adjusted estimates and their precision (eg, 95% confidence interval). Make clear which confounders were adjusted for and why they were included | N/A |
| | | (*b*) Report category boundaries when continuous variables were categorized | N/A |
| | | (*c*) If relevant, consider translating estimates of relative risk into absolute risk for a meaningful time period | N/A |
| Other analyses | 17 | Report other analyses done—eg analyses of subgroups and interactions, and sensitivity analyses | N/A |
| **Discussion** | | | |
| Key results | 18 | Summarise key results with reference to study objectives | N/A |
| Limitations | 19 | Discuss limitations of the study, taking into account sources of potential bias or imprecision. Discuss both direction and magnitude of any potential bias | N/A |
| Interpretation | 20 | Give a cautious overall interpretation of results considering objectives, limitations, multiplicity of analyses, results from similar studies, and other relevant evidence | N/A |
| Generalisability | 21 | Discuss the generalisability (external validity) of the study results | N/A |
| **Other information** | | | |
| Funding | 22 | Give the source of funding and the role of the funders for the present study and, if applicable, for the original study on which the present article is based | 2 |

*Give information separately for cases and controls in case-control studies and, if applicable, for exposed and unexposed groups in cohort and cross-sectional studies.

**Note:** An Explanation and Elaboration article discusses each checklist item and gives methodological background and published examples of transparent reporting. The STROBE checklist is best used in conjunction with this article (freely available on the Web sites of PLoS Medicine at http://www.plosmedicine.org/, Annals of Internal Medicine at http://www.annals.org/, and Epidemiology at http://www.epidem.com/). Information on the STROBE Initiative is available at www.strobe-statement.org.

For peer review only
