# Supplementary material for: Therapies for Long COVID in non-hospitalised individuals: from symptoms, patient-reported outcomes and immunology to targeted therapies (The TLC Study)
Source: BMJ Open. 2022 Apr 25;12(4):e060413. doi: 10.1136/bmjopen-2021-060413 (PMC9044550; doi:10.1136/bmjopen-2021-060413)
Supplement: Reviewer comments [file bmjopen-2021-060413.reviewer_comments.pdf]

PEER REVIEW HISTORY

BMJ Open publishes all reviews undertaken for accepted manuscripts. Reviewers are asked to complete a checklist review form (**http://bmjopen.bmj.com/site/about/resources/checklist.pdf**) and are provided with free text boxes to elaborate on their assessment. These free text comments are reproduced below.

ARTICLE DETAILS

**TITLE (PROVISIONAL)**	Therapies for Long COVID in non-hospitalised individuals - from symptoms, patient-reported outcomes, and immunology to targeted therapies (The TLC Study): Study protocol
**AUTHORS**	Haroon, Shamil; Nirantharakumar, Krishnarajah; Hughes, Sarah; Subramanian, Anuradhaa; Aiyegbusi, Olalekan Lee; Davies, Elin Haf; Myles, Puja; Williams, Tim; Turner, Grace; Chandan, Joht Singh; McMullan, Christel; Lord, Janet; Wraith, David; McGee, Kirsty; Denniston, Alastair; Taverner, Thomas; Jackson, Louise; Sapey, Elizabeth; Gkoutos, George; Gokhale, Krishna; Leggett, Edward; Iles, Clare; Frost, Christopher; McNamara, Gary; Bamford, Amy; Marshall, Tom; Zemedikun, Dawit; Price, Gary; Marwaha, Steven; Simms-Williams, Nikita; Brown, Kirsty; Walker, Anita; Jones, Karen; Matthews, Karen; Camaradou, Jennifer; Saint-Cricq, Michael; Kumar, Sumita; Alder, Yvonne; Stanton, David E.; Agyen, Lisa; Baber, Megan; Blaize, Hannah; Calvert, Melanie

This article was not externally reviewed at BMJ Open. Protocols that have been independently assessed prior to submission to BMJ Open are usually fast-tracked to publication on the grounds that further substantial changes will not be possible. This independent assessment will usually be external, independent review for both a substantial grant award from a non-commercial or government funder and ethics approval.
